# Nucleophagy contributes to genome stability through degradation of type II topoisomerases A and B and nucleolar components

**DOI:** 10.1242/jcs.260563

**Published:** 2023-01-12

**Authors:** Gabriel Muciño-Hernández, Pilar Sarah Acevo-Rodríguez, Sandra Cabrera-Benitez, Adán Oswaldo Guerrero, Horacio Merchant-Larios, Susana Castro-Obregón

**Affiliations:** ^1^ Departamento de Neurodesarrollo y Fisiología, División de Neurociencias, Instituto de Fisiología Celular, Universidad Nacional Autónoma de México, 04510 Mexico City, México; ^2^ Facultad de Ciencias, Universidad Nacional Autónoma de México, 04510 Mexico City, México; ^3^ Laboratorio Nacional de Microscopía Avanzada, Instituto de Biotecnología, Universidad Nacional Autónoma de México, 62210 Cuernavaca, Morelos, Mexico; ^4^ Departamento de Biología Celular y Fisiología, Instituto de Investigaciones Biomédicas, Universidad Nacional Autónoma de México, 04510 Mexico City, Mexico

**Keywords:** Mammalian nucleophagy, Autophagy, DNA damage, Nucleolus, Micronuclei

## Abstract

The nuclear architecture of mammalian cells can be altered as a consequence of anomalous accumulation of nuclear proteins or genomic alterations. Most of the knowledge about nuclear dynamics comes from studies on cancerous cells. How normal healthy cells maintain genome stability, avoiding accumulation of nuclear damaged material, is less understood. Here, we describe that primary mouse embryonic fibroblasts develop a basal level of nuclear buds and micronuclei, which increase after etoposide-induced DNA double-stranded breaks. Both basal and induced nuclear buds and micronuclei colocalize with the autophagic proteins BECN1 and LC3B (also known as MAP1LC3B) and with acidic vesicles, suggesting their clearance by nucleophagy. Some of the nuclear alterations also contain autophagic proteins and type II DNA topoisomerases (TOP2A and TOP2B), or the nucleolar protein fibrillarin, implying they are also targets of nucleophagy. We propose that basal nucleophagy contributes to genome and nuclear stability, as well as in response to DNA damage.

## Teaser

**Summary:** Mammalian cells form nuclear buds and micronuclei containing autophagy proteins in a basal way and in response to DNA double strand breaks, suggesting homeostatic nuclear maintenance by nucleophagy.

## INTRODUCTION

Genome stability is essential for the proper function of the cells, Genome instability is also a common feature of several pathologies primary affecting the nervous, immune and reproductive systems, and it also contributes to neurodevelopmental disorders, neurodegeneration, cancer development and premature aging (Ciccia and Elledge, 2010). From early in the developmental process of organisms, DNA is under constant endogenous challenges, for example when local abundant cell proliferation leads to DNA replication stress. It has been uncovered recently that cells produce DNA breaks as a physiological mechanism. For example, in response to TGFB1-induced epithelial-to-mesenchymal transition (Dobersch et al., 2021; Singh et al., 2015), as well as in active neurons (Madabhushi et al., 2015), DNA double-strand breaks (DSBs) facilitate chromatin opening to initiate transcription of early-response genes. Another source of physiological DSBs is the active recombination that occurs during differentiation of B and T immune cells to produce multiple antibodies and receptors, respectively (Schatz and Ji, 2011). Interestingly, in primary neural stem and progenitor cells, a set of genes related to neuronal function are targets of active DSBs, suggesting that a recombination event similar to that seen in immune system development also occurs in the nervous system (Alt et al., 2017). These active DSBs and their repair could provide a mechanistic explanation for the mosaic nature of the mammalian brain recently described, pointing out that such genome dynamics processes also exist in post-mitotic cells (Rohrback et al., 2018). Additionally, there are exogenous challenges to DNA integrity, such as DNA chemical modifications and DNA breaks caused by reactive oxygen species derived from normal cell metabolism. Exogenous sources of reactive oxygen species come from radiation and chemicals, giving rise to multiple types of DNA modifications. Therefore, DNA integrity needs to be constantly monitored and repaired. Eukaryotic cells have developed a network of intracellular pathways that sense DNA damage, signal to coordinate a cellular response and repair damaged DNA, collectively known as the DNA damage response (DDR). An example of a DNA lesion sensor is ATM kinase, which phosphorylates mediator proteins, such as histone variant H2AX (with the phosphorylated form known as γH2AX) (Ciccia and Elledge, 2010). γH2AX is phosphorylated at serine 139 (Rogakou et al., 1998) at sites nearby to DSBs, which in turn initiates a cascade of DNA repair factor assembly (Ciccia and Elledge, 2010).

The DDR regulates the recruitment of DNA repair molecules suitable to repair particular types of DNA damage. A defective DDR leads to genomic instability manifested either as minor chromosomal alterations or as pronounced chromosomal rearrangements (Langie et al., 2015). Owing to the repetitive nature of ribosomal DNA present in the nucleolus, and its active transcription, this genomic region is especially susceptible to instability. Hence, chromosomal rearrangements of ribosomal DNA is frequently observed in tumor cells (Korsholm et al., 2020). Accumulation of genomic alterations canaffect the nuclear structure, eliciting the extrusion of nuclear content into the cytoplasm, forming nuclear buds and micronuclei. The latter are fragments of chromosomes or whole chromosomes surrounded by nuclear envelope (Fenech et al., 2011; Kisurina-Evgenieva et al., 2016; Rao et al., 2008). The presence of micronuclei contributes to malignant cell transformation (Hintzsche et al., 2017). It is essential to remove micronuclei before their nuclear envelope is damaged, since micronuclear envelope rupture causes gain or loss of genetic material and chromothripsis (extensive chromosome rearrangements confined to one or few chromosomes). Micronuclear envelope rupture also leads to DNA exposure to the cytoplasm, activating the cyclic GMP-AMP synthase (cGAS)-STING pathway, which triggers the innate immune response inducing inflammation (Kwon et al., 2020). In senescent cells, fragments of chromatin containing damaged DNA are expelled from nuclei into the cytoplasm free of nuclear envelope, and are called cytoplasmic chromatin fragments (Ivanov et al., 2013). Cytoplasmic chromatin fragments also trigger inflammation by the activation of the cGAS-STING pathway (Dou et al., 2017).

Gene transcription, replication, recombination etc. generate DNA entanglements (coiling and winding of the DNA double helix), which are resolved by DNA topoisomerases. Among them, type II topoisomerases (TOP2; TOP2A and TOP2B in mammals) catalyze the resolution of DNA entanglements by creating transient DNA DSBs that allow topological changes. During this process, TOP2 binds covalently to the 5′ end in the broken DNA forming a transitory intermediate cleavage complex (TOP2cc). Etoposide is a topoisomerase poison that stabilizes TOP2cc by misaligning DNA ends. This action prevents re-ligation, which results in trapping of TOP2 on DNA termini, generating cytotoxic protein-linked DNA breaks that cells need to eliminate to avoid genome instability (Ashour et al., 2015; Austin et al., 2018).

In mammals, specifically in cancer cell lines, macroautophagy (hereafter autophagy) is activated by genotoxic stress (Chen et al., 2015) and contributes to the removal of extruded nuclear material (Erenpreisa et al., 2011; Rello-Varona et al., 2012; Zhao et al., 2021). Cytoplasmic chromatin fragments in senescent cells are also removed by autophagy (Ivanov et al., 2013). In autophagy-deficient cells, chromosomal abnormalities and deficiencies in DNA damage repair occur (Bae and Guan, 2011; Chicote et al., 2020). Hence, autophagy seems to be protective of the genome, as the activation of different DNA repair pathways triggers autophagy, contributing to resolution of genomic instability (Eliopoulos et al., 2016). The degradation of nuclear components by the autophagic machinery is coined nucleophagy. Recently, the cGAS protein has been proposed to function as a nucleophagy receptor (Zhao et al., 2021).

In this work, we hypothesized that nucleophagy could be a mechanism to maintain nuclear and genome integrity in normal (noncancerous) cells, in response to DNA-damaging agents. We found that primary mouse embryonic fibroblasts (MEFs) developed nuclear buds and micronuclei in response to DSB caused by etoposide. Nuclear alterations contained damaged DNA and TOP2cc (Austin et al., 2018), as well as nucleolar components such as the rRNA 2′-O-methyltransferase fibrillarin. These nuclear alterations were surrounded by the autophagic proteins LC3B (also known as MAP1LC3B) and BECN1, in proximity with lysosomal markers, indicative of their potential elimination by nucleophagy. Inhibition of autophagy reduced the frequency of nuclear buds, suggesting an active role of the autophagic machinery in their formation. Surprisingly, we observed that the number of micronuclei increased in the absence of the autophagic protein ATG4, supporting the notion that buds and micronuclei have different mechanisms of formation. Interestingly, we also observed basal development of nuclear buds and micronuclei in control cells, which were also surrounded by autophagy machinery. Collectively, our data show that nucleophagy contributes to preserve nuclear cell physiology by constantly clearing damaged DNA through nuclear buds and micronuclei elimination, both at basal levels and in response to genotoxic stress.

## RESULTS

### Nuclear buds and micronuclei form in primary fibroblasts and increase with etoposide-induced DSBs

**Fig. 1.**
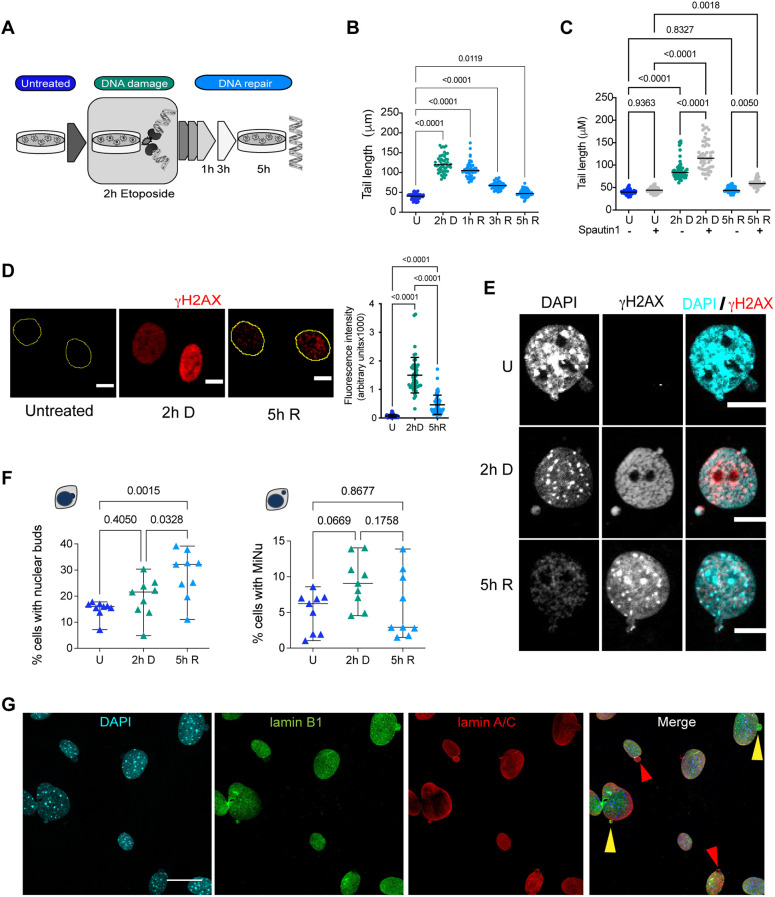
**There is a basal formation of nuclear buds and micronuclei in primary fibroblasts, which increases with etoposide-mediated induction of DSBs.** (A) Workflow for the DNA damage and repair assay. MEFs were exposed to 120 µM etoposide for 2 h to damage DNA (2 h D), then etoposide was removed to allow DNA repair, which was monitored after 1, 3 or 5 h. (B) Quantification of comet tail length (which is proportional to the number of DSBs) in untreated cells (U), after 2 h of etoposide exposure (2 h D), and after 1 h, 3 h or 5 h of etoposide removal (1 h R, 3 h R, 5 h R, respectively). Bars represent median at each time point, statistical significant differences were determined by one-way ANOVA followed by Dunnett's multiple comparison test; adjusted *P*-value is indicated for each comparison. 50 comets were measured in each of three independent experiments. Detailed data are shown in Table S1. (C) Quantification of comet tail length in untreated cells (U), after 2 h of etoposide exposure (2 h D), and 5 h of etoposide removal (5 h R), previously treated for 12 h with vehicle (−) or 10 µM Spautin1 (+). Bars represent median at each time point, statistical significant differences were determined by one-way ANOVA followed by Dunnett's multiple comparison test; adjusted *P-*value is indicated for each comparison. 50 comets were measured in each of three independent experiments. Detailed data are shown in Table S1. (D) DDR followed by the recruitment of γH2AX in untreated (U), damaged (2 h D) or repaired (5 h R) DNA. Yellow contours indicate the nuclei of cells. Scale bars: 10 µm. The fluorescence signal was quantified in 48 cells per experiment in three independent experiments and the mean±s.d. is graphed to the right. Statistical significant difference was determined by One-way ANOVA followed by Tukey′s multiple comparison test; adjusted *P*-value is indicated for each comparison. Detailed data are shown in Table S1. (E) Nuclear buds or independent micronuclei were observed by confocal microscopy in untreated (U), damaged (2 h D) or repaired (5 h R) DNA. DNA damaged marked with γH2AX (red) was found in both buds and micronuclei, mainly when cells were treated with etoposide. DNA was stained with DAPI. Scale bars: 10 µm. (F) Quantification of the percentage of cells with nuclear buds or micronuclei (MiNu) in untreated (U), damaged (2 h D) or repaired (5 h R) DNA. The mean±s.d. of nine independent experiments is graphed. Statistical significant difference was determined by one-way ANOVA followed by Dunnett's multiple comparison test; adjusted *P*-value is indicated for each comparison. For every experiment (represented as triangles) at least 50 cells were counted; detailed data are shown in Table S1. (G) Representative immunofluorescence images from five independent experiments to detect lamin A/C (red) and lamin B1 (green) in MEFs treated with etoposide for 2 h (2 h D). Yellow arrowheads show examples of buds containing both lamin A/C and lamin B1. Red arrowheads show examples of buds containing only lamin A/C. Scale bar: 30 µm.

Given that most of our knowledge about micronuclei formation and elimination comes from studies with cancerous cells, we aimed to study micronuclei formation induced by DSBs, which are the most toxic DNA lesions for cells, in primary cells (Ciccia and Elledge, 2010). Primary MEFs were treated with 120 µM etoposide for 2 h to cause DSBs that were detectable by a neutral comet assay. Mean comet tail length increased from a mean of 39.54±7.594 µm (s.d.) in untreated cells to 122.8±22.08 µm (mean±s.d.; *P*<0.0001) after 2 h of etoposide treatment. To analyze DNA repair, etoposide was removed (see Fig. 1A for experimental design). DSBs were gradually repaired, having shorter comet tails (107.9±8.894 µm, *P*<0.0001) at 3 h after etoposide removal, and becoming almost undetectable after 5 h of recovery (comet tail length 48.33±8.994 µm; *P*=0.0119). 50 comets were measured in each of three independent experiments (Fig. 1B; Fig. S1A; raw data are presented in Table S1). We evaluated the contribution of autophagy to DNA repair in our model. Spautin1 is an autophagy inhibitor that acts by promoting BECN1 and PI3K-III degradation (Liu et al., 2011). We treated cells with Spautin1 for 12 h before etoposide treatment. As can be observed in Fig. 1C, autophagy inhibition increased the level of DSBs produced after 2 h of etoposide treatment from a mean comet tail length of 89.05±22.28 µm in etoposide only cells, to 121.4 µm (±31.91; *P*<0.0001) in Spautin1 plus etoposide cells. In cells pre-treated with Spautin1, DNA repair occurred with a reduction of mean comet tail to 59.77±8.2 µm, although to a lesser extent that when cells were exposed to etoposide only (45.44±6.23 µm), remaining statistically different after 5 h of recovery (*P*=0.005). This observation suggests that autophagy contributes to DNA stability. We then evaluated DDR activation by analyzing the recruitment of the DNA damage marker γH2AX. We observed at 2 h of etoposide exposure abundant γH2AX (a mean±s.d. of 15,002±6223 arbitrary fluorescent units) compared to untreated control cells (675.3±569.3 arbitrary fluorescent units; *P*<0.0001). The level of γH2AX was reduced after 5 h of recovery to 4635±3367 arbitrary fluorescent units (*P*<0.0001) (Fig. 1D; Fig. S1B). Cell viability remained ≥80% during both DNA damage and repair (Fig. S1C). High levels of DNA damage leads to deformations of nuclear architecture and micronuclei formation (Kisurina-Evgenieva et al., 2016; Medvedeva et al., 2007), and the induction of multiple DSBs results in the budding of nuclear envelope and micronuclei formation (Okamoto et al., 2012; Utani et al., 2011). Hence, we analyzed the nuclear structure of MEFs treated with a sublethal dose of etoposide. As expected, we found that etoposide-treated cells bear nuclear protrusions or buds containing damaged DNA, identified by γH2AX. Interestingly, we also found nuclear buds and micronuclei in a subpopulation of control untreated cells (Fig. 1E). This observation implies that cells normally have a basal dynamic formation of nuclear buds and micronuclei, which to our knowledge has not been reported before. Interestingly, although the frequency of nuclear buds gradually increased after DNA damage from 15.09±3.19% (mean±s.d.) in untreated cells to 19.43±7.52% (*P*=0.3362) after 2 h of damage, and then to 28.37±9.06% (*P*=0.001) after 5 h of DNA repair, the frequency of micronuclei also increased after DNA damage from 5.096±2.75% to 9.207±3.46% (*P*=0.0492), but diminished upon DNA repair to 5.98±4.63% (*P*=0.8309) (Fig. 1F). We observed that the cytoplasmic damaged DNA was contained within micronuclei, as they were found surrounded by lamin A/C and lamin B1. In some micronuclei, we observed only lamin A/C (Fig. 1G). The reduction in the frequency of micronuclei once DNA has been repaired suggests that the onset of buds and micronuclei formation upon DNA damage has a similar dynamic, but micronuclei are being actively removed during DNA repair.

### Autophagy is necessary for basal and DSB-induced nuclear bud formation and micronuclei removal in primary fibroblasts

In cancerous cell lines, micronuclei removal is carried out by nucleophagy (Erenpreisa et al., 2011; Rello-Varona et al., 2012). We asked whether basal or DNA damage-induced nuclear buds and micronuclei could also be eliminated by nucleophagy in primary MEFs.

**Fig. 2.**
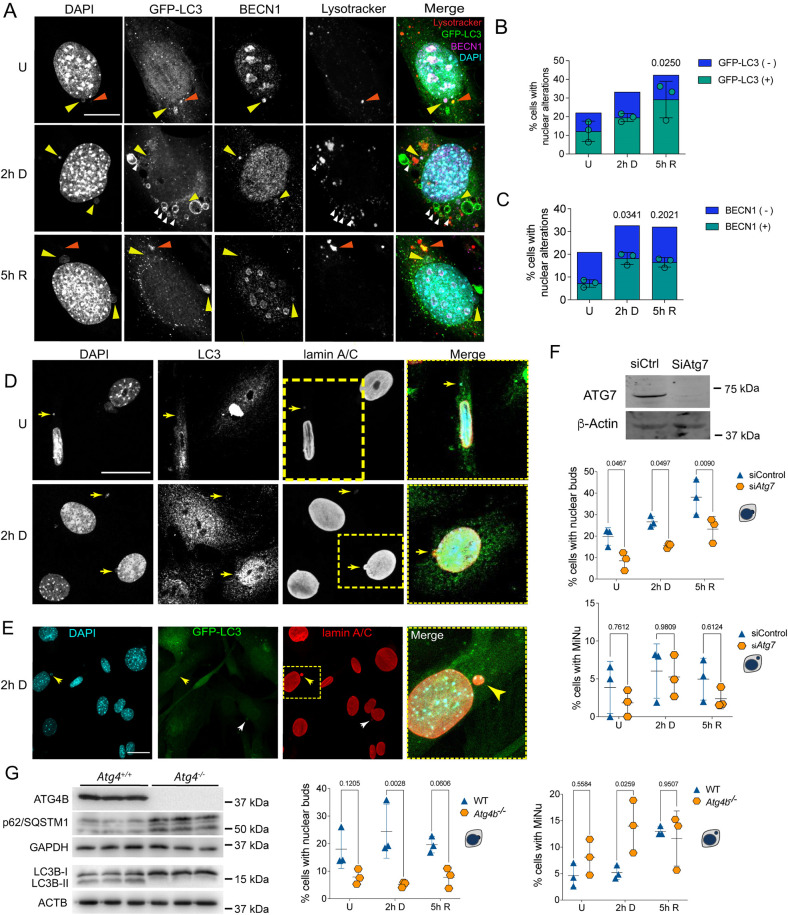
**Nuclear buds and micronuclei are associated with components of different stages of the autophagic pathway.** (A) Representative images of autophagic proteins GFP–LC3 and BECN1 found in nuclear buds (yellow arrowhead) in MEFs that were untreated (U), treated for 2 h with etoposide (2 h D) or after 5 h of DNA repair (5 h R), as used for quantifications shown in B and C. Some micronuclei were contained in autolysosomes, identified by having DNA (DAPI), GFP–LC3 and Lysotracker® staining (orange arrowheads). GFP–LC3-labeled vesicles next to Lysotracker^®^ staining, or with Lysotracker® staining inside, are shown with white arrowheads. Scale bar: 10 µm. (B,C) Percentage of cells with nuclear alterations (nuclear buds and micronuclei). Among nuclear alterations, those containing GFP–LC3 (B) or BECN1 (C) are shown in green, whereas those without GFP–LC3 or BECN1 are shown in blue. Color bars represent the mean of three independent experiments. Green symbols represent the percentage of cells with nuclear alterations containing GFP–LC3 or BECN1; bars represent mean±s.d. The percentage of cells with nuclear buds or micronuclei are shown independently in Fig. S3. At least 50 cells were counted per treatment and experiment, and significant differences were determined by one-way ANOVA followed by a Kruskal–Wallis test; *P-*value is indicated in comparison with untreated samples. (D) Representative images of endogenous LC3B localized in micronuclei surrounded by lamin A/C, and containing DNA detected by DAPI staining (yellow arrows) in MEFs untreated (U) or treated for 2 h with etoposide (2 h D). Yellow squares indicate the magnified areas shown to the right. Scale bar: 30 μm. (E) Representative micronuclei surrounded by lamin A/C containing GFP–LC3 (yellow arrows) in MEFs treated for 2 h with etoposide. Yellow dotted square indicates the magnified area shown to the right. Scale bar: 30 µm. Images in D and E are representative of five repeats. (F) Functional autophagy seems to be necessary to form nuclear buds. MEFs were transfected with siRNA control (siCtrl) or *siAtg7* for 48 h and then treated or not with etoposide for 2 h and left to repair DNA for 5 h [untreated (U), damaged (2 h D) or repaired (5 h R) DNA]. The western blot shows representative level of *Atg7* silencing; β-actin was used as loading control. Whole blots are shown in Fig. S4A. Graphs show the percentage of cells with nuclear buds (top) or micronuclei (MiNu; bottom). For every experiment at least 50 cells were counted by detecting DAPI signal in nuclear alterations in confocal images. The distribution of the data from three independent experiments is graphed (mean±s.d.). Significant differences were obtained by two-way ANOVA analysis, followed by a Sidak's multiple comparison test. Adjusted *P*-values are indicated for each comparison. (G) Functional autophagy seems to be necessary for micronuclei elimination. WT and *Atg4b^−/−^* MEFs were analyzed to evaluate the abundance of nuclear alterations. Western blot demonstrates lack of ATG4B in *Atg4b^−/−^* MEFs, accompanied by an accumulation of p62/SQSTM1 protein and absence of LC3B lipidation (lack of LC3B-II), confirming deficient autophagosome formation. The indicated sizes correspond to the molecular mass markers used for each blot. Whole blots are shown in Fig. S4B. Graphs show the percentage of cells with nuclear buds (left) or micronuclei (right). For every experiment at least 140 cells were counted by detecting the DAPI signal in nuclear alterations in confocal images. The distribution of the data from three independent experiments is graphed (mean±s.d.). Significant differences were analyzed by two-way ANOVA following a Sidak's multiple comparison test; *P*-value is shown for each comparison. Detailed data of every graph are shown in Table S1.

We followed the distribution of GFP-tagged LC3B (GFP–LC3) in nuclear alterations and found that 52.3±8.2% (mean±s.d.) of the nuclear buds and micronuclei contained GFP–LC3 in control cells, 60.3±8.7% after 2 h of DNA damage, and 69.7±17.5% after 5 h of DNA repair (Fig. 2A,B). We also monitored the intracellular distribution of BECN1, another protein required for autophagosome formation (Zeng et al., 2006). Just as with LC3B, we found that 34.5±8.3% (mean±s.d.) of the nuclear buds and micronuclei contained BECN1 in untreated cells, which increased to 55.7±1.9% after DNA damage and slightly decreased to 52.9±10.2% after 5 h of DNA repair (Fig. 2A,C). We also noticed a nuclear enrichment of both GFP–LC3 and BECN1 (a representative wider field is shown in Fig. S2), which agrees with previous observations indicating that autophagy mediates degradation of the nuclear lamina through a direct interaction between LC3B and lamin B1 in proliferating cells, and this interaction helps to translocate lamin B1 into the cytoplasm for its lysosomal degradation during oncogene-induced senescence (Dou et al., 2015). We speculated that LC3B could also contribute to the translocation of nuclear damaged material into the cytoplasm for autolysosomal degradation in primary cells. We analyzed whether nuclear buds and micronuclei were associated with autolysosomes. We found micronuclei containing DNA and LC3B, stained with Lysotracker^®^ (Fig. 2A) and for BECN1 (Fig. S2). Interestingly, we noticed that 22.48% of untreated cells also had nuclear buds and micronuclei containing LC3B and 20.99% had these containing BECN1. This observation suggests that there is a basal level of nuclear dynamics, constantly forming nuclear protrusions and micronuclei, perhaps to eliminate genomic alterations that are frequently produced. We confirmed the micronuclei nature of the cytoplasmic vesicles with DNA and LC3B by detecting lamin A/C (Fig. 2D). To determine a causal role of the autophagic machinery in nuclear bud and micronuclei removal, the expression of *Atg7,* a member of the ubiquitin-like system required for autophagosome elongation (Simon et al., 2017), was silenced by specific siRNA before DNA damage induction. Surprisingly, the percentage of cells with nuclear buds decreased from them being present in 19.8±4.16% (mean±s.d.) of control cells to 8.543±4.25% in *siAtg7* cells. In response to etoposide-induced DSBs, the percentage of cells containing buds dropped from 26.68±2.56% of control cells to 15.56±1.12% of *siAtg7* cells; after 5 h of DNA repair a similar response was observed – the percentage of cells having nuclear buds were reduced from 38.14±8.22% of control cells to 23.28±5.83% of *siAtg7* cells, as shown in Fig. 2F. Interestingly, we also observed a small reduction in the percentage of cells with micronuclei when *Atg7* was silenced, although no statistical significant difference was found. We observed 3.86±3.46% of cells with micronuclei in control cells, and 1.82±1.76% in *siAtg7* cells. When DNA was damaged, we found 6.03±3.59% of cells with micronuclei and 5.25±2.76% of *siAtg7* cells. After 5 h of DNA repair 4.95±2.77% of cells had micronuclei, and 2.38±1.34% in *siAtg7* cells (Fig. 2F). These results suggest that components of the autophagy machinery actively induce the formation of nuclear buds but perhaps does not participate in micronuclei formation to the same extent. To confirm the contribution of the autophagic machinery in formation of nuclear alterations and their elimination, we used *Atg4b^−/−^* MEFs, which have deficient autophagy demonstrated by a lack of LC3B-I conversion to LC3B-II, and an accumulation of the autophagic receptor p62/SQSTM1 (which is degraded by autophagy) (Fig. 2G). Whereas the percentage of control wild-type (WT) MEFs with buds was 17.98±7.02%, in *Atg4b^−/−^* MEFs it was only 7.89±2.77%. After 2 h of DNA damage, 24.51±9.83% of control WT MEFs had buds, whereas just 5.21±1.03% of *Atg4b^−/−^* MEFs had buds. And once DNA was repaired 19.59±2.97% of WT MEFs had buds, whereas only 7.78±3.68% of *Atg4b^−/−^* MEFs had buds. However, when we compared the percentage of cells with micronuclei, we found the opposite, an increase instead of a reduction of micronuclei in the absence of ATG4. We observed 4.63±2.28% of control WT MEFs with micronuclei and 8.11±3.39% in control *Atg4b^−/−^* MEFs. After 2 h of DNA damage 5.18±1.28% of WT MEFs had micronuclei and 13.96±4.98% of *Atg4b^−/−^* MEFs had them. And after 5 h of DNA repair, the percentage of cells with micronuclei was 13±0.88% of WT MEFs and 11.63±5.21% of *Atg4b^−/−^* MEFs (Fig. 2G). A multiple comparison analysis among time points and treatments is shown in Fig. S2.

In summary, we found nuclear buds and micronuclei with markers of different stages of the autophagic pathway, suggesting an active role of autophagy proteins in buds formation, and basal micronuclei removal and during DNA damage.

### Nucleophagy clears topoisomerase cleavage complex and nucleolar fibrillarin

**Fig. 3.**
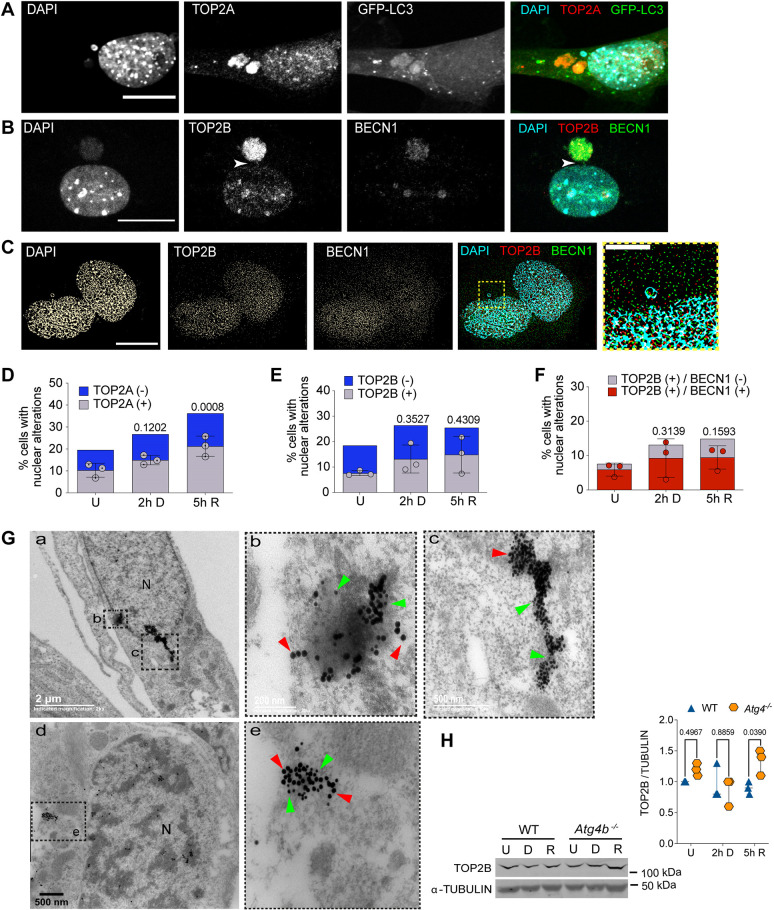
**TOP2cc are targeted for nucleophagic clearance.** (A) Representative confocal image after immunofluorescence staining to detect TOP2A in MEFs expressing GFP–LC3, treated with 120 µM etoposide for 2 h. Scale bar: 20 µm. (B) Representative confocal image after immunofluorescence staining to detect TOP2B and BECN1 in MEFs treated with 120 µM etoposide for 2 h. Scale bar: 20 µm. Arrowheads show a bridge contacting both the main nucleus and a micronucleus containing both TOP2B and BECN1 signals. (C) Representative images obtained by super-resolution microscopy to detect colocalization of DNA and TOP2B (TOP2Bcc) with BECN1 in MEFs after 5 h of DNA repair. Yellow square represents the magnified section presented to the right. Scale bar: 15 µm; magnified section, 5 µm. Images in A–C are representative of three repeats. (D) Percentage of untreated (U), DNA damaged (2 h D) or DNA repaired (5 h R) cells with nuclear alterations (nuclear buds and micronuclei) containing DNA and TOP2A (gray bars). Nuclear alterations without TOP2A are shown as blue bars. The mean±s.d. for three independent experiments (counting at least 50 cells per experiment) are graphed. (E,F) Percentage of cells with nuclear alterations (nuclear buds and micronuclei) containing TOP2B (in E) or TOP2B colocalizing with BECN1 (in F) in untreated MEFs or after DNA damage (2 h D, cells treated with 120 µM etoposide for 2 h) or DNA repair phase (5 h R, cells after 5 h of etoposide removal). At least 50 cells were counted for each experiment. The mean±s.d. of three independent experiments is graphed. In D–F, statistical significance was calculated by two-way ANOVA followed by Dunnett's multiple comparison test; adjusted *P*-values are shown for each comparison. (G) Electron micrographs showing simultaneous detection of LC3B and TOP2B by immunogold labeling. Figures b and c show higher magnification views of the area indicated in a; e shows a higher magnification views of the area indicated in d. Green arrowheads show examples of 15 nm gold particles coupled to secondary antibody to detect TOP2B and red arrowheads point to 25 nm gold particles coupled to secondary antibody to detect LC3B. Images in G are representative of three repeats. (H) Western blot of total extracts from WT or *Atg4b^−/−^* MEFs that were untreated (U), treated for 2 h with etoposide (D) or after 5 h of DNA repair (R) to compare the abundance of TOP2B in the presence (WT) or absence of ATG4 (*Atg4b^−/−^*). α-Tubulin was detected as a loading control. Whole blots are presented in Fig. S4. Graph shows a densitometric analysis of three independent experiments. Statistical significance was determined by two-way ANOVA followed by Sidak's multiple comparisons test. Adjusted *P*-values are shown for each comparison.

Several mechanisms to remove TOP2cc have been observed. For example, TDP2 hydrolyzes the phosphodiester bond between TOP2 and DNA after partial TOP2 degradation. In an alternative mechanism, nucleases remove TOP2 and a fragment of DNA (Ashour et al., 2015; Pommier et al., 2016). We propose that nucleophagy might also contribute to the elimination of these complexes. To analyze this, we studied whether TOP2 were found in nuclear alterations (both nuclear buds and micronuclei) and within autophagosomes. By immunolocalization, we found both TOP2A and TOP2B were present in nuclear alterations containing DNA at a basal level in control cells, which was increased upon etoposide induction of DSBs. We quantified the percentage of cells with nuclear alterations and found that 53±3.14% (mean±s.d.) of them contained TOP2A in untreated cells, compared to 56±2.14% in cells with 2 h of DNA damage and 59±4.61% after 5 h of DNA repair. Similar results were obtained for TOP2B, where 41±1.0% of the cells with nuclear alterations in untreated cells contained TOP2B, 50±5.51% in cells with 2 h of DNA damage and 58±7.18% in cells with 5 h of DNA repair (Fig. 3A–E). We found TOP2A within GFP–LC3-positive micronuclei (Fig. 3A), and TOP2B within micronuclei also containing BECN1 (Fig. 3B). We confirmed by super-resolution microscopy that TOP2B localization was within nuclear alterations containing both DNA and BECN1 (Fig. 3C) in 78±2.9% of the nuclear alterations in untreated cells (Fig. 3F). After etoposide-induced DSBs, we observed that 70±3.4% of nuclear alterations contained BECN1 and TOP2B, which then were reduced to 63±4.3% after DNA repair. We noticed in some cells a bridge joining the main nucleus with micronuclei that contained both TOP2B and BECN1 (Fig. 3B,F). We further demonstrated TOP2B nucleophagic degradation by immunogold colocalization of LC3B and TOP2B observed by transmission electron microscopy. TOP2B was found surrounded by LC3B in transit towards the cytoplasm, confirming the frequent nucleophagic degradation of nuclear alterations (Fig. 3G). To verify an autophagic degradation of TOP2B, we compared by western blotting, the abundance of TOP2B in WT MEFs with *Atg4b^−/−^* MEFs. In spite of it being only a subpopulation of cells that presented micronuclei at the times analyzed, we observed a subtle accumulation of TOP2B in *Atg4b^−/−^* MEFs (Fig. 3H).

**Fig. 4.**
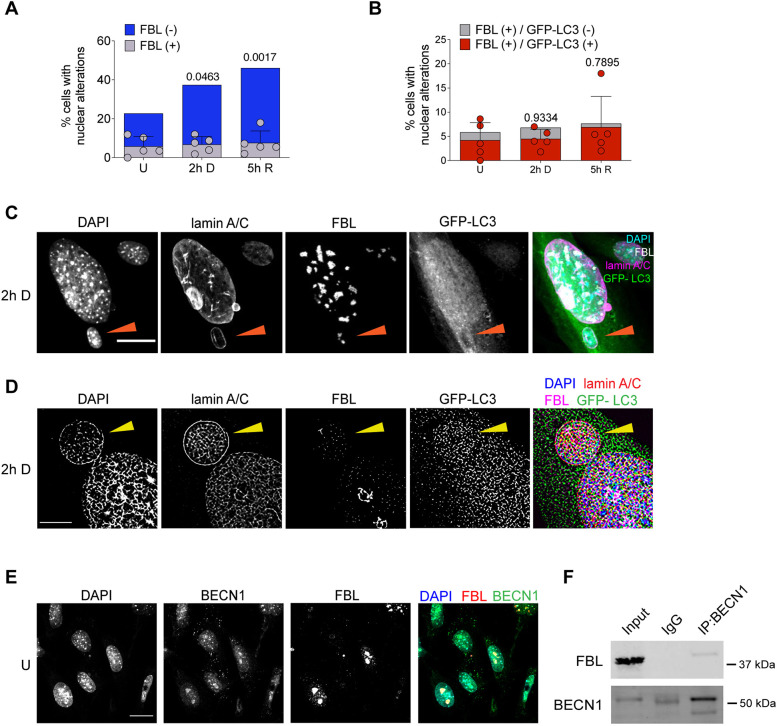
**The nucleolar protein fibrillarin is found in nuclear buds and micronuclei containing autophagic proteins.** (A) Percentage of cells containing nuclear alterations (buds and micronuclei), with (gray bars) or without (blue bars) fibrillarin (FBL) in untreated cells (U), or after etoposide treatment (2 h D) or after 5 h of DNA repair (5 h R). (B) Percentage of cells containing nuclear alterations (buds and micronuclei), with (gray bars) or without (red bars) FBL and GFP–LC3 in untreated cells (U), or after etoposide treatment (2 h D) or after 5 h of DNA repair (5 h R). For A and B, dots represent the mean of each experiment (*n*=5); at least 50 cells were counted per experiment by analyzing DAPI distribution in confocal images. Bars correspond to s.d. A two-way-ANOVA followed by Dunnett′s multiple comparison test of the total number of nuclear alterations. Adjusted *P*-values are indicated for each comparison with control cells. Even though there is a trend to increase nuclear alterations containing fibrillarin upon DNA damage, no statistical significant differences were obtained. Detailed data are presented in Table S1. (C) Representative confocal microscopy images showing fibrillarin (FBL) in a micronucleus containing DNA stained with DAPI and surrounded by lamin A/C (arrowhead) and GFP–LC3, in cells treated with etoposide for 2 h. Scale bar: 20 µm. (D) Representative super-resolution microscopy images of the same experiment described in C. Scale bar: 5 µm. (E) Representative confocal microscopy images showing the concurrent distribution of FBL and BECN1 in control cells. Scale bar: 20 µm. (F) Immunoprecipitation (IP) of BECN1, and western blot to detect FBL or BECN1 as indicated, from total protein extract of untreated cells. IgG was used as control of FBL-specific interaction with BECN1. Whole blots are shown in Fig. S4C. Images in C–E are representative of three repeats. Blots in F are representative of two repeats.

To maintain genome stability in the ribosomal DNA domain is particularly challenging given that it is located in the nucleolus. The nucleolus is a subnuclear compartment with a high density of nucleic acids and proteins that creates a distinct environment that limits the accessibility of DNA repair factors (Korsholm et al., 2020). We considered that nucleosomal damage could also be removed by expelling nucleolar damaged material into the cytoplasm to be a nucleophagy target. In teratocarcinoma cells, nucleolar aggresomes increase in response to etoposide exposure, and are transported to the cytoplasm where they are surrounded by the autophagic machinery (Salmina et al., 2017). We looked for the presence of fibrillarin (FBL), a nucleolar marker, in micronuclei and nuclear buds in primary MEFs, treated or not treated with etoposide. As shown in Fig. 4A, fibrillarin was found in micronuclei and nuclear buds in 5.86±5.03% (mean±s.d.) of untreated cells, indicating a basal level of nucleolar material exclusion from the nucleus. In this set of experiments, 16.9±9.97% of control cells had nuclear alterations (nuclear buds and micronuclei) without fibrillarin. Interestingly, in cells treated with etoposide for 2 h we observed only a slight increase to 6.8±4.03% of cells having nuclear buds and micronuclei with fibrillarin, whereas the number of cells with other nuclear lesions increased to 30.6±4.2%. Similarly, the proportion of cells having fibrillarin in nuclear lesions after 5 h of DNA repair only increased to 7.66±6.08%, whereas the percentage number of cells having nuclear buds and micronuclei without fibrillarin increased to 38.42±9.3%. These results suggest that nucleolar components are constantly sent out of the nucleus as a homeostatic process, and not significantly in response to etoposide-induced DSBs. We confirmed, by both confocal and super-resolution microscopy, the micronuclear compartment of the cytoplasmic fibrillarin, finding it with DNA surrounded by lamin-A/C (Fig. 4C,D). We then analyzed whether nuclear buds and micronuclei containing fibrillarin were also a target of autophagic proteins. As shown in Fig. 4B–D, we detected GFP–LC3 in 72±3.61% of the nuclear alterations containing fibrillarin in untreated cells, and in 65.7±1.97% of cells with 2 h of DNA damage. Noticeably, after 5 h of DNA repair 90.33±6.36% of the fibrillarin-containing nuclear perturbation had GFP–LC3 (Fig. 4B). Given that in the above experiments, we had observed a subnuclear localization of BECN1 resembling nucleolar structures (see Figs 2A and 3B), and considering the basal extrusion of fibrillarin found here, we speculated that BECN1 could be located at the nucleolus in control cells. We observed by immunolocalization a similar distribution of fibrillarin and BECN1 (Fig. 4E) and confirmed by co-immunoprecipitation that BECN1 and fibrillarin are in the same complex (Fig. 4F). Taken together, these observations suggest that nucleolar components are potential targets of autophagic degradation.

## DISCUSSION

Several studies have shown that autophagy contributes to genome stability by different mechanisms; for example, it elevates the level of DNA repair proteins of both the homologous recombination (HR) and non-homologous end joining (NHEJ) pathways, and enhances DNA damage recognition to be repaired by nucleotide excision repair (Lin et al., 2015; Liu et al., 2015). Additionally, BECN1 interacts directly with TOP2B, which leads to the activation of DNA repair proteins, and the formation of NR and DNA-PK repair complexes (Xu et al., 2017). Cytoplasmic elimination of nuclear lamina components has been observed in cells with oncogenic insults, with LC3B interacting directly with both lamin-associated domains on chromatin and lamin B1 and lamin A/C to be targeted for lysosomal degradation (Dou et al., 2015; Lenain et al., 2015; Li et al., 2019). These observations lead to the conclusion that nucleophagy contributes to tumor suppression. Here, we describe that BECN1, together with LC3B, could have a pivotal role in control (untreated) cells and in cells with etoposide-induced DSBs, integrating the DNA repair machinery with the autophagy machinery. Autophagic proteins seem to promote the formation of nuclear buds, given that the percentage of cells with this type of nuclear alterations diminished in cells with *Atg7* silenced and in cells lacking ATG4B. Interestingly, the formation of micronuclei seems to be mechanistically different to that of buds, as the inhibition of autophagy did not reduce the percentage of cells with micronuclei. By contrast, cells with micronuclei accumulated in *Atg4b^−/−^* MEFs in comparison with WT MEFs, both at the basal level and after DNA damage (Fig. 2F,G). A difference in the biogenesis of buds and micronuclei has been previously suggested, studying cells cultured under strong stress conditions that induce DNA amplification, as well as in cells under folic acid deficiency. While interstitial DNA without telomere was more prevalent in buds than in micronuclei, telomeric DNA was more frequently observed in micronuclei (Fenech et al., 2011).

There is also variability in the composition of the micronuclear envelope. By a careful analysis of the protrusions and micronuclei formed, we observed in some cases only one type of nuclear lamin, either lamin A/C or lamin B1, was present, whereas in other cases, both nuclear lamins were present (Fig. 1F). The different micronuclear envelope composition is probably related to different DNA damage or different DNA structures affected that lead to their formation. Others have also identified structural differences in micronuclei envelope with a variable presence of lamin B1, which has been linked to different abilities to replicate (Okamoto et al., 2012) or repair (Terradas et al., 2012) the genome, by affecting the recruitment of proteins required in those processes. These differences in the envelope composition could affect their nucleophagic removal.

The identification of TOP2 and fibrillarin in micronuclei containing autophagic proteins led us to propose their elimination by nucleophagy. In support of this notion are the following findings: (1) both BECN1 and LC3B were detected in micronuclei in acidic vesicles (labeled with Lysotracker), which is indicative of the autolysosomal nature of the cytoplasmic compartment (Fig. 2A); (2) TOP2B was found by electron microscopy exiting the nucleus surrounded by LC3B in some cells (Fig. 3G); and (3) TOP2B accumulated in cells lacking ATG4, as expected if it is degraded by autophagy (Fig. 3H).

Along with the results presented here, the damage inflicted by etoposide was detected with a wide γH2AX signal, which implies a huge amount of TOP2cc that has to be removed. We propose that nucleophagy contributes, with other mechanisms previously described to eliminate peptides or the whole TOP2 protein, free and complexed with DNA, such as the proteasome, phosphodiesterases and nucleases. To our knowledge, this is the first report of TOP2 degradation by autophagy. In support of our finding, it has been described that there is a decrease in TOP2A when cancerous cells are grown under glucose deprivation (Alchanati et al., 2009), an autophagy-inducing condition (Klionsky et al., 2021).

We noticed that in control cells some micronuclei did not have γH2AX or were not stained with DAPI, suggesting that the nuclear material to be extruded did not always contain DSB. Perhaps other types of damaged DNA are extruded, or it is conceivable that nuclear buds and micronuclei could be formed for a proteostatic function, not necessarily involving DNA damage elimination. The recruitment of multiple molecules for DNA repair into the nucleus could trigger an imbalance in nuclear proteostasis, and the proteasome could become overloaded. Even though it has been shown that the proteasome degrades TOP2 (Mao et al., 2001) and fibrillarin (Chen et al., 2002), our findings suggest that nucleophagy could have a collaborative role with the proteasome, contributing to protect both genome integrity and nuclear morphology.

An outcome of the overloaded activity of ubiquitin-proteasome system (UPS) is the accumulation and aggregation of polyubiquitylated proteins as aggresomes (Latonen et al., 2011). This occurs in the cytoplasm but also in the nucleoplasm, specifically at nucleoli, where under different stress conditions (heat shock, acidosis or genotoxic insults) proteins, RNA and conjugated ubiquitin accumulate (Jacob et al., 2013; Latonen, 2011, 2019). For example, under DNA damage, an early and transient nucleolar accumulation of paraspeckle proteins (Moore et al., 2011) (paraspeckles are nuclear subcompartments which function as a reservoir for splicing factors; Nunes and Moretti, 2017) and E2F1 occurs, affecting the structure and function of the nucleolus (Jin et al., 2014). The final destiny for aggresomes is not totally understood, but it has been suggested they persist until UPS degradative capacity is recovered (Latonen et al., 2011). Another possibility is that the aggresome is cleared by autophagy to promote genome stability and cell viability (Salmina et al., 2017). In this work, we observed nuclear alterations containing fibrillarin in control cells, the levels of which slightly increased during DNA damage and repair, although without a statistical significant difference (Fig. 4C,D). A proportion of such nuclear alterations, mainly nuclear buds, had nuclear lamin A/C and GFP–LC3. It suggests that nucleolar components are targeted for autolysosomal degradation. Consequently, nucleophagy could be a mechanism to alleviate basal nucleolar stress.

In summary, the data presented support the contribution of autophagic proteins to extrude damaged DNA, TOP2cc and Fibrillarin from the nucleus, preventing nuclear distortions and genotoxic stress. Insufficiencies on autophagy imply a risk of genomic instability, which in turn could drive the cell into a senescent or malignant state.

## MATERIALS AND METHODS

### Animals and cell culture

CD1 and GFP–LC3 (C57BL/6J) (Mizushima et al., 2004) animals were obtained from the animal house of the Institute of Cellular Physiology (IFC) at the National Autonomous University of Mexico (UNAM). Mutant mice deficient in autophagy-related 4B (*Atg4b^–/–^* mice) (Marino et al., 2010) were obtained from the animal house of the National Institute of Respiratory Diseases of Mexico (INER). Animals were housed at 22°C in 12 h light–12 h dark cycle with *ad libitum* access to water and food. Mice used in the present study were handled and cared according to the animal care and ethics legislation. All procedures were approved by the Internal Committee of Care and Use of Laboratory Animals of the IFC (IFC-SCO174-21).

All the experiments were done with mouse embryonic fibroblasts (MEFs) at cell passage 4 or 5. MEFs from WT CD1, GFP–LC3 transgenic mice or *Atg4b^–/–^* mice were obtained at embryonic day (E)13.5 according to the standard protocol (Xu, 2005). Lack of contamination with mycoplasma was tested in every batch using the VenorGeM Mycoplasma Detection Kit (Sigma-Aldrich MP-0025, St Louis MO, USA), following the procedure indicated by the provider. MEFs were grown in Dulbecco's modified Eagle's medium plus GlutaMAX^TM^, 10% FBS and 100 U/ml penicillin-streptomycin. Media and supplements were from GIBCO® Life Technologies, Grand Island, NY, USA. Culture conditions consisted of a humidified 5% CO_2_ atmosphere at 37°C. DNA damage was induced by incubating cells with etoposide (Etopos® injectable solution, Lemery, Mexico City, Mexico) at 120 μM for 0.5, 1 or 2 h. Then etoposide was removed and cells were washed twice with 1× PBS and incubated for 1, 3, 5 or 24 h in fresh medium.

#### siRNA transfection

WT MEFs were transfected using Lipofectamine 2000 (Invitrogen, Carlsbad, CA, USA) according to manufacturer's instructions. Briefly, 5×10^4^ cells/well were seeded into 12-well plates 24 h before transfection and using antibiotic-free medium. For each well, 20 pmol siRNA and 3 µl Lipofectamine were mixed and added for 6 h. After that fresh antibiotic-free medium was added and cells were incubated for 48 h. SMARTpool siRNA ATG7-FITC was from Dharmacon (Lafayette, CO, USA). Control siRNA targeted a region of a Luciferase-coding gene.

### Neutral comet assay

The DSBs were detected with a neutral comet assay. Briefly, ∼100 cells/µl were resuspended in PBS and mixed at a 1:5 ratio with 0.75% low-melting point agarose (Bio-Rad Certified™ Low Melt Agarose #1613112, Bio-Rad, Hercules, CA, USA) at 37°C. Then with the help of a coverslip, ∼50 µl of the previous mix was spread on glass slides pre-coated with 1% normal-melting point agarose (Bio-Rad Certified™ PCR Agarose #1613104, Bio-Rad). The slides were incubated first at 4°C for 2 min and then for an extra 10 min at room temperature. After the removal of coverslip, each slide was sequentially covered and incubated for 60 min with pre-chilled lysis solution and then with unwinding buffer at 4°C. Next electrophoresis was performed with slides by applying 25 V for 20 min. After that, slides were incubated in neutralization buffer for 10 min, repeating this steps three times. Next, SYBR green (solution 1:10,000 in 1× PBS, SYBR^TM^ green I Nucleic Acid Gel Stain, Invitrogen, Eugene, OR, USA) was used to stain DNA. Lysis solution was 0.03 M EDTA, 1% SDS; unwinding and electrophoresis buffer: 60 mM Tris-HCl pH 9.0, 90 mM acetic acid, 2.5 mM EDTA; neutralization buffer was 500 mM Tris-HCl pH 7.5.

To visualize the comets (DNA), a Nikon Eclipse Ti-U microscope with 20× objective and the NIS Elements BR software (Nikon Instruments Inc®, NY, USA) was used to acquire and analyze images. For analysis, the length and area of broken DNA were determined by processing 50 comet images for each treatment.

### Immunofluorescence

The day before treatments, cells were grown on coverslips at a density of 2.5×10^4^ cells/cm^2^ on 12-well plates. After treatments, cells at room temperature were fixed with 4% paraformaldehyde for 30 min, then washed with PBS, permeabilized for 5 min with PBS with 0.5% Triton X-100 and blocked for 1 h with 4% BSA in PBS. Coverslips were incubated overnight at 4°C with primary antibody (diluted in 2% BSA in PBS). The next day, after than removing the primary antibody and a wash with PBS, Alexa Fluor-conjugated secondary antibodies (diluted 1:500 in 2% BSA in PBS) (Life Technologies, OR, USA) were added and incubated for 1 h at room temperature. Finally, nuclei were stained with DAPI (1 μg/ml) for 10 min.

Primary antibodies used for immunofluorescence were: mouse anit-γH2AX (1:1000, ab26350, abcam, Cambridge, MA, USA), mouse anti-Lamin A/C (1:1000, sc-376248, Santa Cruz Biotechnology, Dallas, TX, USA), rabbit anti-Lamin B1 (1:1000, ab16048, abcam), rabbit anti-LC3B (1:1000, 2775S, Cell Signaling Technology, Beverly, MA, USA), rabbit anti-Beclin1 (1:100, sc-11427, Santa Cruz Biotechnology), mouse anti-TOP2A (1:100, sc-365916, Santa Cruz Biotechnology), mouse anti-TOP2B (1:100, sc-25330, Santa Cruz Biotechnology), rabbit anti-Fibrillarin (1:1000, ab5821, abcam).

Secondary antibodies used were: goat anti-mouse IgG (H+L) Alexa Fluor A594 (1:500) (A11032), goat anti-rabbit IgG (H+L) Alexa Fluor A594 (1:500) (A11037) Goat anti-mouse IgG (H+L) Alexa Fluor A488 (1:500) (A11029), Goat anti-rabbit IgG (H+L) Alexa Fluor A488 (1:500) (A11029) All secondary antibodies were from Life Technologies, OR, USA except donkey anti-rabbit IgG (H+L) Alexa Fluor 647, which was from Jackson ImmunoResearch Laboratories, PA, USA.

### Immunoblotting analysis

Cells were lysed using a buffer with 62.5 mM Tris-HCl pH 6.5, 2% SDS and 2 mg/ml protease inhibitor 18 (Complete, Roche Molecular Diagnostics, Pleasanton, CA, USA). Between 30 to 120 µg of protein lysates were separated by SDS-PAGE and then transferred to polyvinylidene fluoride (PVDF) membranes. Following a 1 h blocking step, membranes were incubated overnight with primary antibodies. Secondary antibody IRDye® 680RD goat anti-rabbit (925-68071, LI-COR, Lincoln, NE, USA) or IRDye® 800CW goat anti-mouse (925-32210, LI-COR) were added at 1:5000 dilution in Tris-buffered saline containing 0.1% Tween 20 (TTBS). Scanning was performed using the Odyssey® IR scanner, and image acquisition and analysis were performed using Odyssey® Image Studio software 5.2.5 (LI-COR). Blocking solution consisted of 3% nonfat dry milk (Blotting-Grade Blocker, cat. #170-6404, Bio-Rad) in TTBS.

Primary antibodies used were: mouse anti-γH2AX (1:1000, 26350, abcam), rabbit anti-LC3B (1:1000, L7543 , Sigma-Aldrich, St Louis, MO, USA), rabbit anti-ATG7 (1:1000, 2631, Cell Signaling Technology), rabbit anti-β-actin (1:10,000, C4, sc-47778; Santa Cruz Biotechnology), mouse anti-α-tubulin (1:10,000, 3873, Cell Signaling Technology), rabbit anti-ATG4B (1:1000, A2981, Sigma-Aldrich), rabbit anti-p62/SQSTM1 (1:1000, P0068, Sigma-Aldrich) and mouse anti-GAPDH (1:1000, sc-47724, Santa Cruz Biotechnology).

Whole images of blots are presented in this work are shown in Fig. S4.

### Confocal imaging

All images were collected as *Z*-stacks with an LSM800 (Zeiss, Oberkochen, Germany) confocal microscope using 40×/1.3 or 63× oil immersion objectives with 1 Airy unit aperture of pinhole. Samples were excited with 405 nm, 488 nm, 561 nm and 640 nm laser lines. CZI files obtained with ZEISS ZEN software and images of *Z*-projection were processed in Fiji (imageJ) software.

### Immunoelectron microscopy

Cells were fixed with 3% glutaraldehyde. Following fixation, dehydration was performed in an ethanol gradient (30%, 40%, 50%, 60%, 70%, 80%, 90% and 100% ethanol) at 4°C. Then, the cells were embedded in a LR White resin and polymerization was carried out at 50°C. Ultrathin sections of 70–80 nm were cut from the polymer using an Ultracut-Recheirt-Jung microtome and placed on nickel grids for immunogold assay.

The thin sections were washed twice for 2 min with deionized water and two times with PBS with 0.005% Tween 20. Sections were then incubated for 30 min with the blocking solution (50 mM glycine, 0.005% Tween 20, 0.01% Triton X-100 and 0.1% BSA in PBS) (Rosas-Arellano et al., 2016). After blocking, sections were incubated with the rabbit anti-LC3B primary antibody (1:500, MBL PD014, Nagoya, Japan). After rinsing three times in PBS with 0.005% Tween 20, the sections were incubated overnight at 4°C with the secondary antibody (1:20). Samples were washed three times with PBS with 0.005% Tween 20 and post-fixed in 2% glutaraldehyde in PBS for 10 min. The sections were then rinsed with distilled water twice for 5 min and contrasted with 2% uranyl acetate, rinsed with water, dried and observed under a JEOL JEM 1200 EXII electron microscope.

Secondary antibodies used were: donkey anti-rabbit IgG (H&L) conjugated to 25-nm gold particles (#25708 Aurion, Electron Microscopy Science, PA, USA) and donkey anti-mouse IgG (H&L) conjugated to 15-nm gold particles (#25817 Aurion, Electron Microscopy Science).

### Super resolution microscopy

Super resolution microscopy imaging was performed at the National Laboratory for Advanced Microcopy (LNMA) of UNAM. Immunofluorescence samples were imaged on a Nanoimager-S (Oxford Nanoimaging Ltd) using widefield fluorescence excitation. Samples were excited by alternating laser illumination with a 405 (DAPI), 473 (Alexa Fluor A488, GFP) and 561 (Alexa Fluor A594) laser lines. Detection of the signal was achieved via an 100×, 1.4 NA, oil-immersion objective (Olympus) and an sCMOS Hamamatsu Orca Flash 4.0 V3 using an embedded image splitter for dual-channel fluorescence acquisition. Imaging time was 33 ms, effective pixel size at object plane was 117 nm. Subdiffraction images were obtained via SRRF, a multi-frame super-resolution microscopy approach which gathers nanoscopic information from the statistical analysis of sequences of images collected at the same imaging plane (Gustafsson et al., 2016). Each super resolution image was derived by the analysis of serial stacks of 100 images collected per fluorescence excitation channel. Each serial stack was drift corrected and analyzed using the NanoJ-core and NanoJ-SRRF plugins of FIJI/Image J (Laine et al., 2019). Parameters used for SRRF computation were ring radius 0.5, radiality magnification 10, axes in ring 8 parameters, Temporal Analysis: AVG. The rest of the parameters were left as the recommended default values (Laine et al., 2019).

### Immunoprecipitation

The immunoprecipitations were carried out using µMACS^TM^, Protein A/G MicroBeads MultiMACS^TM^ Protein A/G kit (MACS molecular; Milteyi Biotec, Auburn, CA), following the manufacturer's instructions. Briefly, cell lysates were mixed with 1 µg of monoclonal antibody and 50 µl of Protein G MicroBeads and incubated for 30 min on ice. Proteins complexed with antibodies and magnetic beads were passed over a separation column coupled with a magnetic field and then eluted from the column. Finally, immunoprecipitated proteins were analyzed by western blotting.

### Statistical analysis

Graphs and data analysis were performed with GraphPad Prism 9 (GraphPad Software Inc. La Jolla, CA, USA). Different tests to determine statistically significant differences were applied as indicated in every figure. Raw data for each figure are detailed in Table S1. Multiple comparison analysis among all treatments are shown in supplementary figures indicated for each experiment.

## Supplementary Material

10.1242/joces.260563_sup1 Supplementary information
