# Supplementary material for: Nucleophagy contributes to genome stability through degradation of type II topoisomerases A and B and nucleolar components
Source: J Cell Sci. 2023 Jan 12;136(1):jcs260563. doi: 10.1242/jcs.260563 (PMC10112964; doi:10.1242/jcs.260563)
Supplement: Supplementary information [file joces-136-260563-s1.pdf]

FIGURE S1

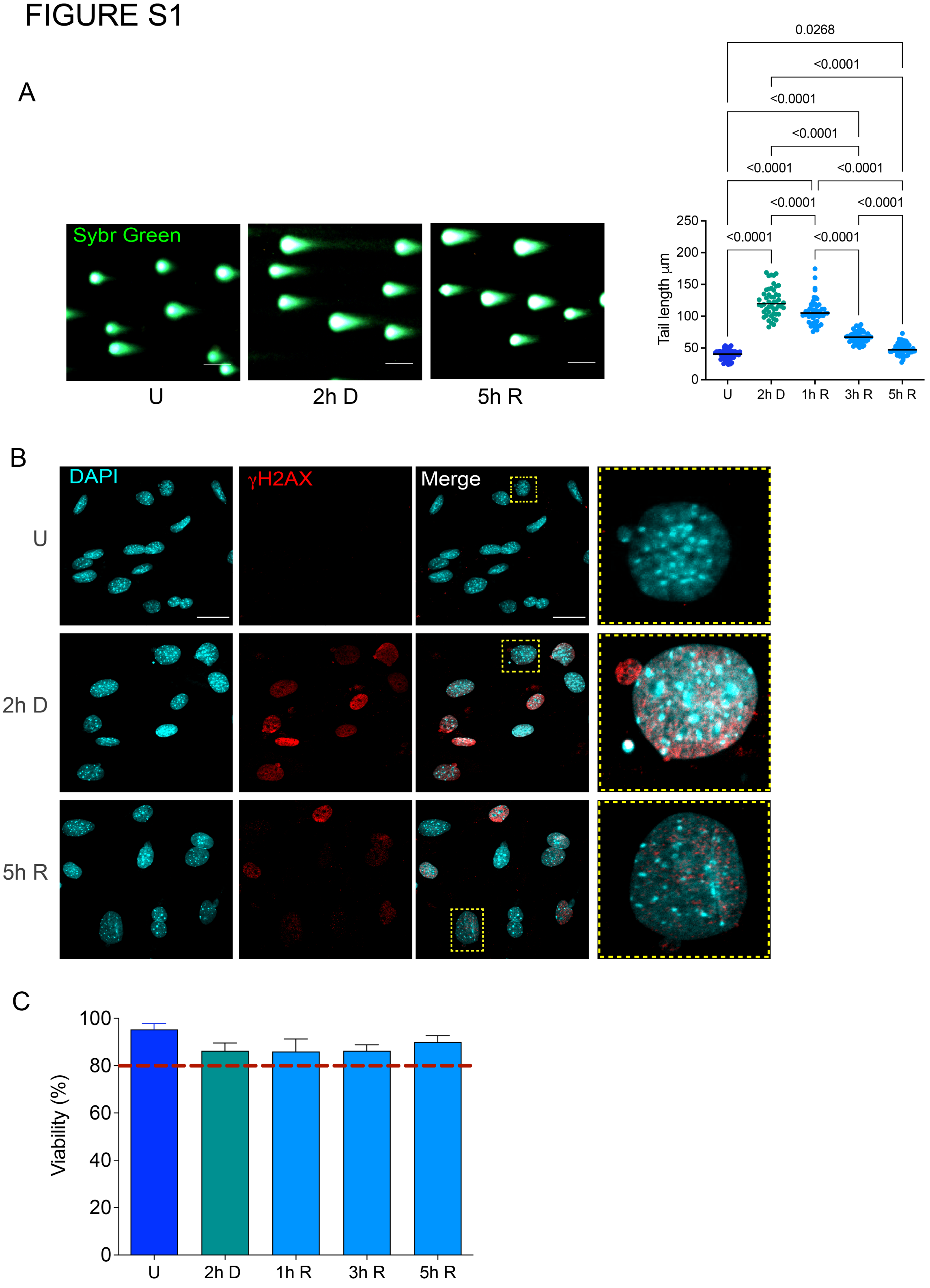

Journal of Cell Science • Supplementary information

Fig. S1. Etoposide treatment in primary MEFs causes DSB, DDR response and increases nuclear alterations. A. Representative images of Comet assay used to quantify data resented in Figure 1B, without treatment (U), after 2h of Etoposide treatment (2h D) and after DNA repair (5h R). DNA was stained with Sybr Green®. Scale bar is equivalent to 100 μm. **B.** DDR was monitored by the immunodetection of γH2AX (in red) at the same time points as in **A**. DNA was stained with DAPI. Scale bar is equivalent to 30 μm. **C.** 2h of Etoposide treatment is sub-lethal. Cell viability was determined by Trypan blue exclusion in MEFs untreated (U), treated with Etoposide for 2 h (2h D) or at the indicated times after Etoposide removal (1hR, 3h R, 5h R). Data are presented as mean ± SD from three independent experiments. Red dashed line indicates 80% of the cell viability.

Journal of Cell Science • Supplementary information

FIGURE S2

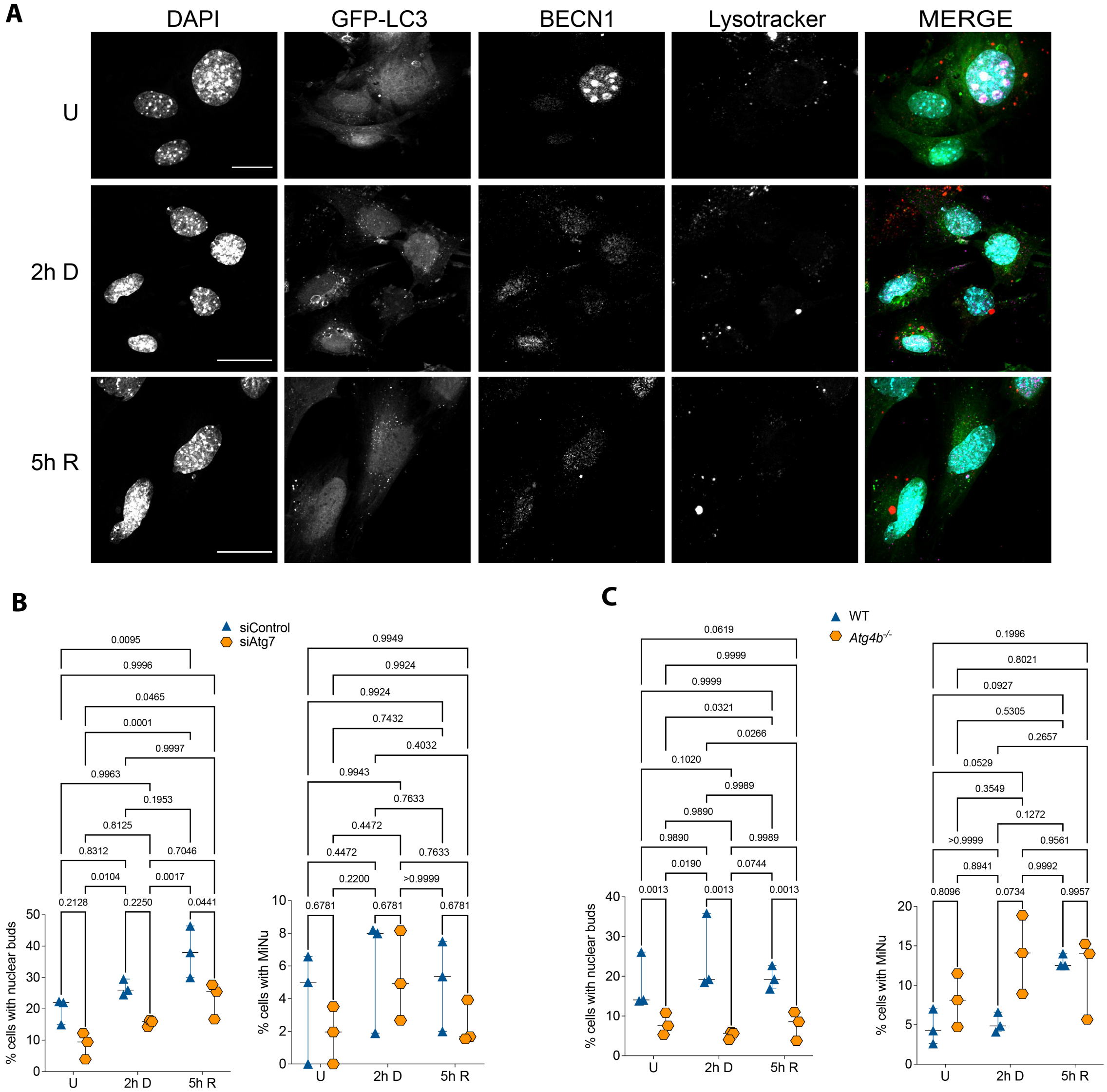

Fig. S2. A. Representative images of whole cells with micronuclei containing autophagic proteins GFP-LC3 and BECN1 in MEFs untreated (U, scale bar represents 10 μm), treated for 2 h with Etoposide (2h D) or after 5h of DNA repair (5h R) corresponding to data presented in Figure 2A. Scale bar corresponds to 30 μm. **B-C**, 2way Anova followed by Tukey´s multiple comparison analysis among all treatment and time points of the experiments described in Fig 2 F-G. Adjusted P values are indicated.

Journal of Cell Science • Supplementary information

FIGURE S3

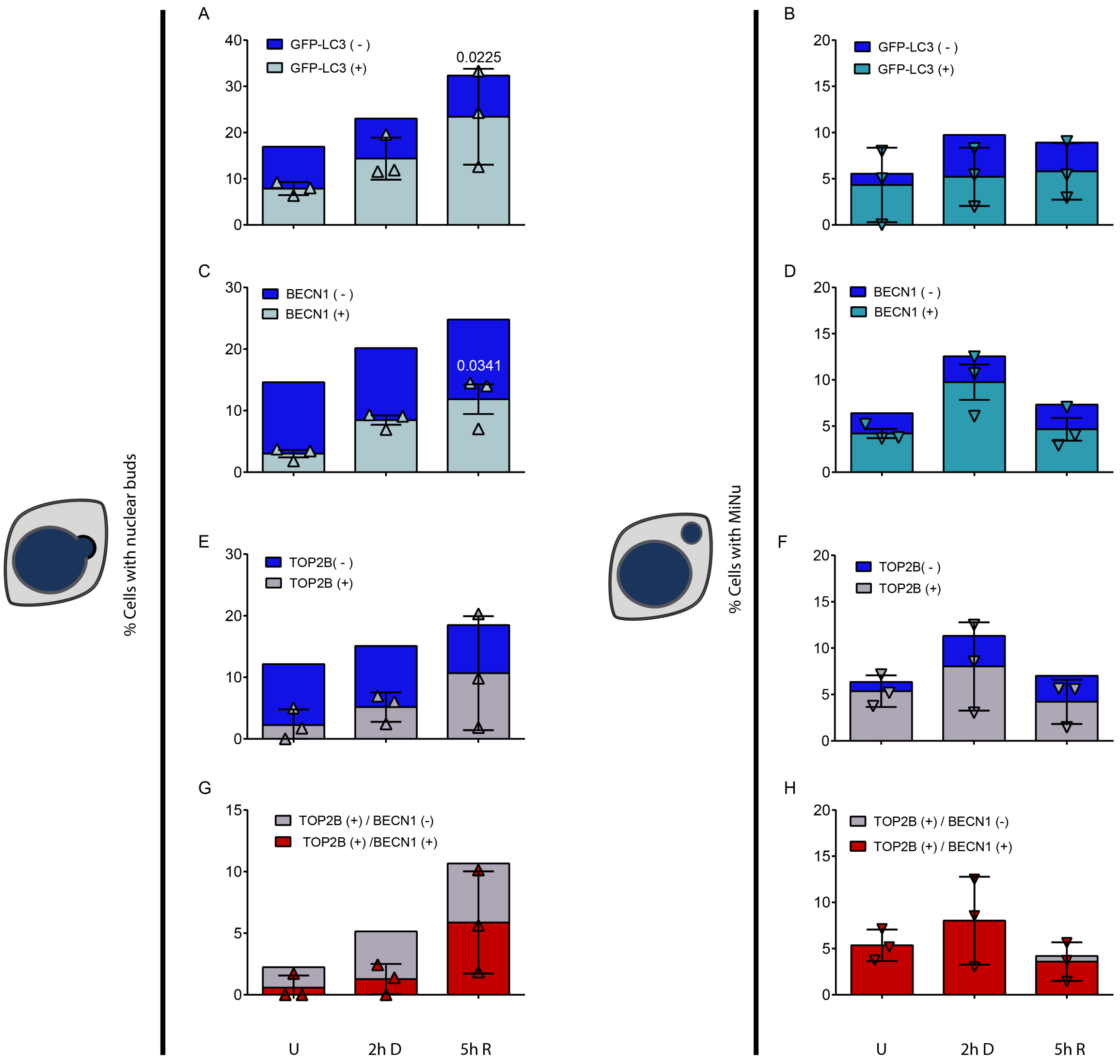

Fig. S3. Nuclear alterations contain autophagic markers and TOP2B. A, B, C and D. Graphs complimentary to Fig 2B and Fig 2C showing the differential quantification of nuclear buds and micronuclei containing GFP-LC3 or BECN1. **E** and **F**. Graphs supplementary to Fig 3E, showing quantification of cells having nuclear buds or micronuclei containing or not TOP2B. **G and H**, percentage of cells with nuclear buds or micronuclei containing TOP2B and co-localizing or not

with BECN1. U, untreated MEFs; 2h D, cells treated with 120 mM Etoposide for 2h; 5h R, cells after 5h of Etoposide removal. At least 50 cells were counted for each experiment. The mean ± SD of three independent experiments is graphed. Statistical significance was calculated by Two-way ANOVA followed by Dunnet´s multiple comparison test; statistical significant P values are shown in comparison with untreated cells. Triangles represent the result of each experiment of the indicated proteins identified.

FIGURE S4

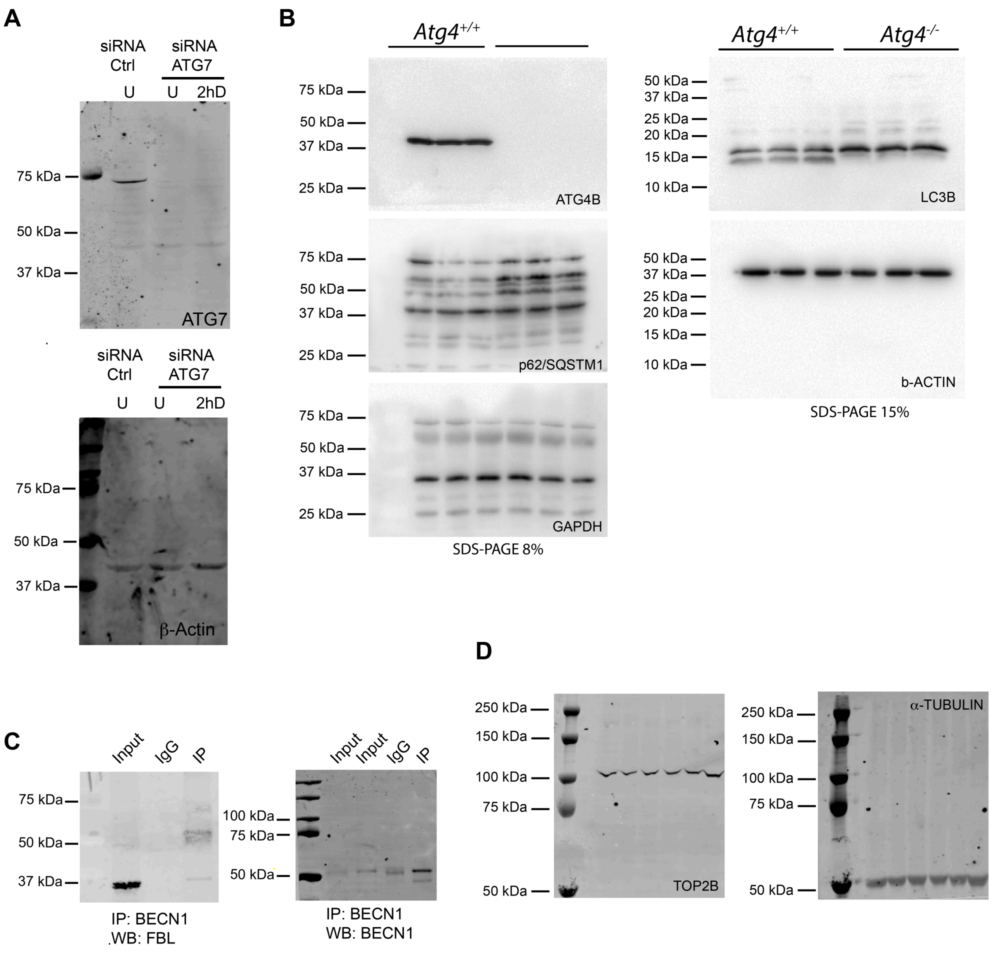

Fig. S4. Blot transparency. A. Whole membranes of Western blots to detect ATG7 shown in Figure 2F. MEFs were transfected with *siRNA-Atg7* or control siRNA for 48h and then treated (2h D) or not (U) with Etoposide for 2h before total protein extraction. β-actin was detected as loading control. **B.** Whole membranes of the Western blots presented in Figure 2G. Total protein extract from WT and *Atg4b*$^{-/-}$ MEFs were analyzed by SDS-PAGE on gels of the indicated acrylamide percentage. **C.** Whole membranes of the Western blots presented in Figure 4F. Total protein

extracts from control MEFs were immunoprecipitated with anti-BECN1 antibody and developed by Western blot for the indicated proteins. **D**. Whole membranes of the Western blots presented in Figure 3H. Total protein extract from WT and *Atg4b*$^{-/-}$ MEFs were analyzed by WB to detect TOP2B. α-TUBULIN was detected as a loading control.

Table S1. Raw data.

Click here to download Table S1

Journal of Cell Science • Supplementary information
