# Supplementary material for: Nucleophagy contributes to genome stability through degradation of type II topoisomerases A and B and nucleolar components
Source: J Cell Sci. 2023 Jan 12;136(1):jcs260563. doi: 10.1242/jcs.260563 (PMC10112964; doi:10.1242/jcs.260563)
Supplement: Supplementary file 1 [file joces-136-260563_review_history.pdf]

Journal of Cell Science
Review COMMONS

Nucleophagy contributes to genome stability through degradation of type II topoisomerases A and B and nucleolar components

Gabriel Muciño-Hernández, Pilar Sarah Acevo-Rodríguez, Sandra Cabrera-Benitez, Adán Oswaldo Guerrero, Horacio Merchant-Larios and Susana Castro-Obregón
DOI: 10.1242/jcs.260563

Editor: Maria Carmo-Fonseca

Review timeline

Submission to Review Commons:	21 August 2022
Original submission:	21 August 2022
Editorial decision:	21 September 2022
First revision received:	22 November 2022
Accepted:	24 November 2022

Reviewer 1

Evidence, reproducibility and clarity

This paper examines the formation and repair of micronuclei in non-cancerous cells, specifically in mouse embryonic fibroblasts. This work was performed completely in culture and used a combination of western blot, confocal and superresolution microscopy to assess the contents of micronuclei over a repair period of 5 hours after 2 hours of induction of double strand breaks by treatment with etoposide. The authors found that the bodies colocalised with LC3, Beclin 1 and lysosomes suggestive of autophagy. However no evidence of autophagic flux has been demonstrated.

Major issues are as follows:

Figure 2
A - Any sense of the autophagic flux? LC3B - I and LC3B - II seem to be in equal quantities most of the time. Maybe using the tandem LC3 in this system could provide further insight. Also remove the violin plots from this graph and from G and H, as there are too few data points.
B. Can you reduce the brightness in the merge image, as I cannot see DAPI nor a convincing Beclin-1/LC3 co-localisation.
F. Although the data is convincing, It would be clearer if the brightness of the merge image was reduced.
G. Is the significant result the difference between 5h R Control si and 5h R Atg7? if so, there is no significant change in micronuclei as the same time point, can you explain this disconnect? are the buds being degraded prior to becoming micronuclei?

Figure 3
A - nice microscopy showing the co-localisation of TOP2A and LC3-GFP. I'm interested in DAPI being on some bodies and not others. Do you have any sense of the dynamics of this? G - c shows a strand of mostly TOP2B coming from the nucleus. Is there any evidence that this occurs using either confocal microscopy or super resolution approaches. Could you try Z-stack to find these?

Figure 4
C - is there a significant increase in FBL negative bodies, this would make sense if FBN is being degraded in the micronuclei during the repair process

© 2022. Published by The Company of Biologists under the terms of the Creative Commons Attribution License (https://creativecommons.org/licenses/by/4.0/).

D. Would it be possible to increase the n of these experiments to confirm either no change in FBL/LC3 co-loc, or evidence of increase?
Minor issues:

Figure 4 and 5 legends are in a different font.

Significance

There is little specific data on the role of autophagy in clearing micronuclei in cancer cells, so this may be suggestive of a new mechanism that occur during normal cellular homeostasis. There are known links between lamin A defects and the formation of micronuclei, but not explicitly that the micronuclei are also Lamin A positive. it is likely that analogous processes occur in both cancer and non-cancer, so the impact of these data is not clear to me. This paper may be of interest to researchers interested in nuclear structure and DNA damage, but based on the data presented the significance is limited.

I don't have sufficient expertise to evaluate the super resolution microscopy beyond assessing the images.

Reviewer 2

Evidence, reproducibility and clarity

Peer review of the manuscript with the number RC-2021-01181 by Muciño-Hernandez G et. al. at Review Commons and with the tittle "Nucleophagy contributes to genome stability 1 though TOP2cc and nucleolar components degradation"

1. Summary

Muciño-Hernandez G et. al. show in this manuscript that mouse embryonic fibroblasts (MEFs) have basal levels of nuclear buds and micronuclei, which are indicators of genomic DNA damage. These basal levels of nuclear buds and micronuclei in MEFs increased after Etoposide treatment, which is known to induce DNA Double stranded Breaks (DSD).
Interestingly, the nuclear buds and micronuclei co-localize with makers for nucleophagy (BECN1 and LC3) and acidic vesicles, suggesting that they are cleared by nucleophagy. The authors propose that basal levels of nucleophagy clear basal levels of genomic DNA damage that occurs as result from DNA-dependent biological processes in the cell nucleus, thereby contributing to nuclear stability of MEFs under physiological conditions. These basal levels of nucleophagy increase after the action of factors that induce DNA damage and nuclear stress. The concepts proposed by Muciño-Hernandez G et. al. are novel, since most of the current published data on nucleophagy related to DNA damage have been obtained under pathological conditions, e.g. implementing cancer cells.

The authors use in their manuscript various molecular biology techniques to obtain data that support their claims, including Western Blot analysis of protein extracts from MEFs, immunostaining on MEFs and neutral comet assays, complemented with state of the art imaging techniques, such as confocal microscopy, immunoelectron microscopy and super resolution microscopy. The quality of the data is sound. The structure of the manuscript support the understanding of the reader. However, I would like to suggest several improvements that will help to increase the quality of the manuscript, in order that fits to the standards of articles recently published in journals affiliated to Review Commons, such as the Journal of Cell Biology, the EMBO Journal or eLife.

2. Major comments

2.1 The authors have to improve the description of the results. Especially the description of those Figure panels containing plots that were generated using data from several experiments has to be improved.

© 2022. Published by The Company of Biologists under the terms of the Creative Commons Attribution License (https://creativecommons.org/licenses/by/4.0/).

One example is the description of the Figure 1D, which is in the lanes 137-151 of the current version of the manuscript. Whereas the authors describe in lanes 137-147 observations related to representative pictures of confocal microscopy after immunostaining presented in Figure 1D (left), the description of the quantification from 9 independent experiments presented in the plots in Figure 1D (right) comes relatively short in lanes 147-150 without mentioning any of the values implemented for creating the plots.

"Interestingly, while the frequency of nuclear buds gradually increased after DNA damage and during DNA repair, the frequency of micronuclei also increased after DNA damage, but diminished upon DNA repair."

The other plots presented in the different figure panels across the manuscript are described in a similar manner. I would like to suggest to the authors to improve their manuscript by including during the description of their results the values that were implemented for the degeneration of the plots presented in the manuscript. For example, in the specific case of Figure 1D above:

"Interestingly, the percentage of MEFs with nuclear buds gradually increased from XY% ({plus minus} XY SD) in control non-treated (Ctrl) MEFs to XY% ({plus minus} XY SD; P=XY) after 2 h Etoposide-induced DSB in MEFs and XY% ({plus minus} XY SD; P=XY) after DNA repair take place in MEFSs 5 h upon stop of Etoposide treatment (Figure 1D, right). In contrast, the percentage of MEFs with micronuclei significantly increased from XY% ({plus minus} XY SD) in Ctrl MEFs to XY% ({plus minus} XY SD; P=XY) after 2 h Etoposide- induced DSB, whereas it was reduced to XY% ({plus minus} XY SD; P=XY) 5 h after stop of Etoposide treatment (Figure 1D, right)."

Descriptions of the plots as mentioned above will make the text more intuitive for the reader, and they will make possible to read the Results Section without switching to the Figure Legends or the Material and Methods Section or to Supplementary Files. Even though the representative pictures from different microscopy techniques presented in the manuscript are of good quality and support the claims of the authors, it is important to mention that the quantifications presented in the plots demonstrate the statistical significance of these representative pictures. Thus, the authors should consistently include in the manuscript during the description of theirs results all the information (mean values, standard error of the means, P values, n values, etc.) that support their interpretation of the results and demonstrate the statistical significance of their claims.

2.2 Following a similar line of argumentation as in the previous point, the authors should provide as Supplementary Material an Excel file containing a statistical summary, including all statistical relevant information from each one of the plots presented in each Figure panel, such as n values, P values, Test implemented, values used for the plots, numbers of experiments, etc. The information could be organized in the Excel file in different data sheets according to the Figure panels, in order that the reader can easily navigate through the data. In the current version of the manuscript, one cannot find the values used for the generation of the plots presented in the manuscript in any of the submitted files.

3. Minor comments

3.1 In general, prior studies were appropriately referenced. Only few references has to be added.

Line 48: Add to the already included reference "Dobersch et al., 2021" also the reference Singh et al., 2015 PMID 26045162.

Line 53: Add the corresponding reference after the word "respectively".

Line 82: Add the corresponding reference after the word "them".

Line 125: Add the corresponding reference after the word "cells".

Line 130: The expression "...by analyzing the recruitment of the phosphorylated histone γH2AX..." is the first time that the authors mention in the manuscript the DNA damage maker γH2AX. I suggest that is better introduced as " ... by analyzing the recruitment of the DNA damage marker

© 2022. Published by The Company of Biologists under the terms of the Creative Commons Attribution License (https://creativecommons.org/licenses/by/4.0/).

γH2AX (histone variant H2A.X phosphorylated a serine 139, Rogakou EP, et al., 1998, PMID 9488723) to DSB sites."

Line 199: Add the corresponding reference after the word "formation".

Line 205: Add the corresponding reference after the word "cells".

3.2 The use of the English language is appropriate throughout the manuscript. However, there are minor errors in the use of punctuation marks, in the use of prepositions and typos. I will list some of them below. However, I would like to recommend that manuscript is corrected by an English native speaker.

Line 41: "...and reproductive systems; genome instability also..." the semicolon can be replaced by a period.

Line 43: "Since early in development DNA is under constant endogenous..." between "development" and "DNA" there should a comma.

The sentence in lanes 53-55 has to be rephrased.

Lines 62-63: the expression "...throughout life." should be substituted.

Line 70: The abbreviation "rDNA" has to be explained the first time that is used.

Lines 81-82: It has to be explained for the scientist that is not specialized in the field of nucleophagy, how the integrity of the genome is threatened by micronuclei and nuclei- derived material.

Lines 106-110: The sentence is long. It would be easier to understand for the reader if this sentence is divided into two sentences.

Lines 121-122: The subtitle should be rephrased.

Lines 132-138: The sentence is long. It would be easier to understand for the reader if this sentence is divided into two sentences, e.g. with a period before the word "hence".

Lines 143-144: "... in a subpopulation of healthy, untreated cells...". The interpretation of "healthy" might be subjective. I would like to suggest substituting in the complete manuscript the word "healthy" by "control".

Line 163: The abbreviation for γH2AX was already introduced in line 130.

Line 182: A comma after "cell lines" is missed.

Line 183: delete "either".

Lines 190-194: The sentence is long. It would be easier to understand for the reader if this sentence is divided into two sentences, e.g. with a period after the word "decreased" in line 191.

Line 218: I assume that instead of "bus", it should be "buds".

Line 220: I assume that instead of "iRNA", it should be "siRNA". In addition, it is the first time that the abbreviation is used. Thus, I suggest introducing it as "...was silenced by specific small interfering RNA (siRNA) previous to ..."

Line 327: delete the word "chronic".

Line 344: I assume that instead of "(figures 4C)", it should be "(Figure 4D)".

© 2022. Published by The Company of Biologists under the terms of the Creative Commons Attribution License (https://creativecommons.org/licenses/by/4.0/).

3.3 The structure of the Figures is ok for the peer review process and it might be optimized during editing of the manuscript. Nevertheless, I would like to suggest to the authors to increase the lettering size throughout all the figures. It will make the figures more intuitive.

Significance

4. Significance
The work presented by Muciño-Hernandez G et. al. will be clearly a significant contribution to the scientific community working on autophagy, DNA damage repair and cancer, among others. It will be of interest to a broad spectrum of scientists, as I will elaborate in the following lines. The authors propose that MEFs have basal levels of genomic DNA damage under physiological conditions, which are cleared by basal levels of nucleophagy. On one hand, these findings are in line with various publications demonstrating that DNA-dependent biological processes in the cell nucleus, such as transcription, replication, recombination, and repair, involve intermediates with DNA breaks that may compromise the integrity of DNA. Thus, there must be mechanisms that ensure the integrity of the genome during these processes under physiological conditions, one of them seems to be nucleophagy. This perspective might explain the fact that proteins and histone modifications that were initially characterized during DNA repair also play a role during transcription, recombination, and replication. For example, phosphorylated H2AX at S139 (?H2AX) is often used as a marker for DNA-DSB [PMID 9488723]. However, accumulating evidences suggest additional functions of this histone modification [PMIDs 19377486; 22628289; 23382544]. In addition, McManus et al. [PMID 16030261] analyzed the dynamics of γH2AX in normal growing mammalian cells and found γH2AX in all phases of cell cycle with a maximum during M phase, suggesting that γH2AX may contribute to the fidelity of the mitotic process, even in the absence of ectopic- induced DNA damage. Further, Singh et al [PMID 26045162] and Dobersch et al [PMID 33594057] report that γH2AX plays a role in transcriptional activation in response to TGFB-signaling. Moreover, classical DNA-repair complexes have been linked to DNA demethylation and transcriptional activation [PMIDs 17268471; 28512237; 25901318], and DNA-DSB is known to induce ectopic transcription that is essential for repair, supporting a tight mechanistic correlation between transcription, DNA damage, and repair [PMID 24207023]. Perhaps, the authors might consider introducing several of the aspects and the citations written above into the Discussion section of the revised version of their manuscript. On the other hand, most of the published data related to nucleophagy have been obtained from cancer cells. Muciño-Hernandez G et. al. obtained their data implementing MEFs to demonstrate that the proposed mechanisms take also place under non-pathological conditions, what is one of the novel aspects of the present work.

I hope that my suggestions help the authors to improve their manuscript, thereby reaching the standards of manuscripts recently published in journals affiliated to Review Commons AND increasing the impact of their contribution to the scientific community.

Reviewer 3

Evidence, reproducibility and clarity

In this manuscript, Muciño-Hernández and colleagues suggest that basal formation of nuclear buds and micronuclei increases in primary mouse embryonic fibroblasts following etoposide-induced double strand breaks (DSBs). The study combines the use of biochemical methodologies with confocal and super resolution microscopy in an effort to explore the contribution of nucleophagy to genome stability. The authors provide evidence that autophagy is induced upon etoposide treatment. They detected GFP-LC3 and BECN1 signals in nuclear buds and micronuclei even in untreated control and to a higher extent in etoposide-treated cells. Then, the authors examined whether nucleophagy is required for the removal of nuclear buds and micronuclei, by treating fibroblasts with control and Atg7 siRNA.
The authors claim that the percentage of cells with micronuclei or nuclear buds decrease upon Atg7 knockdown, suggesting that components of the autophagy machinery induce the formation of these nuclear abnormalities. Moreover, Type II DNA Topoisomerases (TOP2A and TOP2B) and the ribosomal protein fibrillarin were detected in nuclear buds and micronuclei in fibroblasts treated or not with etoposide. Again in this case, GFP-LC3 was detected in fibrillarin-containing nuclear alterations. Based on these observations, the authors suggest that nucleophagy contributes to the

© 2022. Published by The Company of Biologists under the terms of the Creative Commons Attribution License (https://creativecommons.org/licenses/by/4.0/).

elimination of chromosomal fragments or nucleolar bodies exiting the nucleus under DNA damage - inducing conditions. Specifically, they propose a key role for nucleophagy in maintaining genome stability by eliminating Type II DNA Topoisomerase cleavage complex (TOP2cc) and nucleolar components such as fibrillarin.

While it seems that there is a relationship between nuclear-extruded TOP2 with endogenous BECN1 and GFP-LC3 suggesting autophagic engagement, inconsistencies of fluorescent images between different figures indicate possible technical problems/limitations (please see specific comments, below), compromising authors' claims. LC3 immunoblotting and GFP-LC3 localization results appear over-interpreted (comments below). Neither TOP2 nor Fibrillarin have been shown to be actual autophagic substrates. Also, the link between genomic stability, micronuclei formation and autophagy has been previously reported (Zhao et al., PMID: 33752561).

An additional major concern is relates to nucleophagy being a selective type of autophagy. As such it requires efficient recognition and sequestration of the nuclear material destined to be degraded. Cargo specificity is mediated by receptor proteins, but no evidence for such receptors is provided in this study. Moreover, there is no real mechanistic insight on how nucleophagy mediates genome stability and how this can be interpreted in terms of cell survival under physiological and stress conditions. In other words, the biological significance of the findings presented has not been addressed.

Specific comments are summarized below:

The authors suggest that autophagy is induced after etoposide treatment and during the DNA repair process. However, the Western blot presented in Fig. 2A is not convincing and quantification does not support a significant autophagy induction in any of these cases.
Autophagy appears to be induced 1h after etoposide removal, as evidenced LC3II/LC3 I increase (Fig. 2A and S2A). Nevertheless, all these changes should be more rigorously assessed.

Line 190 and Fig. 2A: It is totally unclear whether "autophagy activation" takes place during the two waves described. There is no LC3B-I to LC3B-II conversion to initially suggest "autophagy activation". It rather suggests that autophagy is stalled. Fig. 2F shows that GFP-LC3 is strongly fluorescent into the lysotracker-stained lysosomes, further pointing to possible functional or technical problems.

Fig. 2B and Sup. Fig. 2B: BECN1 staining looks problematic. There is extreme BECN1 accumulation in the nucleus. Are those nuclear patterns of endogenous BECN1 and GFP-LC3 normal (see also minor comment 6 and 7)? Is there literature supporting such a distribution? It is hard to imagine how BECL1 is implicated in a (here hypothetical) nuclear lamina degradation event driven by LC3-lamin B1 direct interaction (Dou et al., 2015).
BECL1 is an upstream to LC3 component and is a subunit of the PI3K complex catalyzing the local PI3P generation. The above should cause recruitment of the downstream autophagic machinery. Other subunits of the same complex or downstream effectors should be identified at the same spots to support authors' claims. U, 2h D and 5h R images of whole cells are necessary. The authors should also provide representative images of cells under different conditions i.e. control, etoposide-treatment and during DNA repair. Along similar lines, untreated control cells are not included in Fig. 2E and F. These images are needed for a better comparison between normal and DNA damage-inducing conditions.

The authors state that autophagy is required for nuclear buds and micronuclei formation. However, the data shown in Fig. 2G and H are hardly convincing given that the statistical difference between cells treated with control and Atg7 siRNA is not strong (for example, *p?0.5, 5h after etoposide removal). To provide further support to this notion, they should use cells from autophagy defective mutants and examine the appearance of nuclear abnormalities across different conditions compared to control cells.

Lines 223-228: The role of autophagic machinery in the formation of nuclear buds is not supported and furthermore hard to conceptualize. How the components of autophagy are implicated during the nuclear buds and micronuclei formation? Colocalization of autophagic proteins might mean that autophagy is engaged at some point after or during the above formation. The causal, mechanistic

© 2022. Published by The Company of Biologists under the terms of the Creative Commons Attribution License (https://creativecommons.org/licenses/by/4.0/).

and temporal aspects of the above budding and nucleophagic events need experimental support and/or more accurate interpretation.

The authors claim that nucleophagy eliminates topoisomerase cleavage complex because TOP2A and TOP2B appear to more extensively co-localize with GFP-LC3 and BECN1 after etoposide-induced DSBs. However, the quantification presented in Fig. 3D-F to support this statement does not, in general, show a statistically significant difference in fibroblasts across different conditions (normal, etoposide treatment, etoposide removal). Why would BECLIN colocalise with TOP2B in Figure 3g, given that beclin is involved in the initiation process?

Fig. 4A and B: There is no enrichment of GFP-LC3 in "the nuclear alterations containing Fibrillarin" as stated in lines 341-343 comparing to the rest of the cellular GFP fluorescence. Moreover, there is no statistical significance in Fig. 4C and D measurements limiting the safety of authors' conclusions in lines 341-346.

Lines 368-370: As discussed by the authors and reported in previous publication (Xu et al., 2017), "BECN1 interacts directly with TOP2B, which leads to the activation of DNA repair proteins, and the formation of NR and DNA-PK repair complexes", independent of its role in autophagy. Currently, there are no rigorous findings supporting the contribution of BECN1 (as a functional constituent of the core autophagic machinery) to nuclear damaged material extrusion (lines 382-384).

Lines 435-441 and Fig. 5: The current findings do not support the proposed model. It is hard to support and conceptualize the statement "proteasome and nucelophagy function in a dynamic way inside the nucleus". In Fig. 5, LC3 appears to decorate inner nuclear membrane and probably to interact with some of the other proteins depicted, which is misleading. Beclin-1 appears to interact with Fibrillarin (Nucleolus).

Most of the differences in Sup. Fig. 3 lack statistical significance compromising the authors' claims.

Many conclusions are drawn by colocalisation-immunofluorescence analysis. Co-immunoprecipitation experiments should also be performed to show that TOP2B and fibrillarin interact with LC3/autophagic machinery. Additionally, colocalisation analysis should be performed using tools such as Pearson's correlation and is an initial indication of nucleophagy. In the case of fibrillarin, immunofluorescence images do not indicate colocalisation, they need to be repeated. Measurement of LC3/fibrillarin positive puncta should be performed, under basal conditions, genotoxic, and nucleolar stress under control and Atg7 knockdown conditions. Moreover, if nuclear proteins described are substrates of autophagy, then their levels would decrease upon autophagic induction i.e. starvation or in this case DNA damage and nucleolar stress. Thus, western blot analysis of relative protein levels can be performed.

Endogenous LC3 nuclear buds should also be detected to verify nucleophagy as GFP-LC3 has been shown to aggregate, causing artifacts under certain conditions.

Minor comments

In the Discussion section, the paragraph focused on the role of the ubiquitin-proteasome system is not substantiated by the data presented in the manuscript. Along similar lines, formation of aggresomes following etoposide treatment and their subsequent removal has not been monitored.

Western blots of better quality should be provided with assigned markers of protein size.

There are several language errors in the text that need to be corrected. Several sentences are too long and confusing or must be re-phrased. For example, see the lines: 123-125, 209- 210,212, 218,221-222.

Fig. 1B. Place "μm" into parenthesis.

Sup. Fig. 1B: Replace "gH2AX" with "?H2AX".

Fig. 1D: Separate DAPI and ?H2AX channel images would be informative.

© 2022. Published by The Company of Biologists under the terms of the Creative Commons Attribution License (https://creativecommons.org/licenses/by/4.0/).

Fig. 2E: Enlarged separate DAPI, GFP-LC3 and lamin A/C channel images would be informative.

Line 218: Replace "bus" with "buds".

Fig. 2B, 2E, 2F, 3A and probably Sup. Fig. 2B represent MEFs treated for 2h with etoposide. The pattern of GFP-LC3 in 2B looks extensively nuclear and almost absent from cytoplasm.

In addition, Fig. 2B and 3B represent MEFs treated for 2h with Etoposide. The pattern of endogenous BECN1 in Fig. 2B looks extensively nuclear and almost absent from cytoplasm. In Fig. 3B the pattern is notably different.

Sup. Fig. 2C: Index box is not properly aligned. Lines 154, 343 and 837: Replace "DBS" with "DSB". Fig. 4 panels are not clearly cited at the text.

Line 220: siRNA

Lines 373-374: References "Lenain et al., 2015" and "Li et al., 2019" are missing.

Lines 400-401 and 407: Probably the second "Latonen, 2011" reference needs "et al".

Line 427: Do authors refer to Fig. 1E rather than Fig. 2B?

Line 434: Correct "clearance" spelling.

Significance

The authors suggest that nucleophagy contributes to the elimination of chromosomal fragments or nucleolar bodies exiting the nucleus under DNA damage -inducing conditions. Specifically, they propose a key role for nucleophagy in maintaining genome stability by eliminating Type II DNA Topoisomerase cleavage complex (TOP2cc) and nucleolar components such as fibrillarin.

However, neither TOP2 nor Fibrillarin have been shown to be actual autophagic substrates. Also, the link between genomic stability, micronuclei formation and autophagy has been previously reported (Zhao et al., PMID: 33752561).

Original submission

First decision letter

MS ID#: JOCES/2022/260563

MS TITLE: Nucleophagy contributes to genome stability though TOP2cc and nucleolar components degradation

AUTHORS: Gabriel Mucino-Hernandez, Pilar S Acevo-Rodriguez, Sandra Cabrera, Adan Oswaldo Guerrero, Horacio Merchant-Larios, and Susana Castro-Obregon
ARTICLE TYPE: Research Article

We have now reached a decision on the above manuscript.

To see the reviewers' reports and a copy of this decision letter, please go to: https://submit-jcs.biologists.org and click on the 'Manuscripts with Decisions' queue in the Author Area. (Corresponding author only has access to reviews.)

As you will see, the reviewers raise a number of substantial criticisms that prevent me from accepting the paper at this stage. Please address these issues as thoroughly as possible. In

© 2022. Published by The Company of Biologists under the terms of the Creative Commons Attribution License (https://creativecommons.org/licenses/by/4.0/).

particular, you should perform quantification of the gH2AX staining in Fig. 1. Otherwise, I consider that no further experiments are required.

Please ensure that you clearly highlight all changes made in the revised manuscript. Please avoid using 'Tracked changes' in Word files as these are lost in PDF conversion.

I should be grateful if you would also provide a point-by-point response detailing how you have dealt with the points raised by the reviewers in the 'Response to Reviewers' box. Please attend to all of the reviewers' comments. If you do not agree with any of their criticisms or suggestions please explain clearly why this is so.

Reviewer 1

Advance summary and potential significance to field

There is little specific data on the role of autophagy in clearing micronuclei in cancer cells, so this may be suggestive of a new mechanism that occur during normal cellular homeostasis. There are known links between lamin A defects and the formation of micronuclei, but not explicitly that the micronuclei are also Lamin A positive. it is likely that analogous processes occur in both cancer and non-cancer, so the impact of these data is not clear to me. This paper may be of interest to researchers interested in nuclear structure and DNA damage

Comments for the author

The manuscript is signficantly improved with the suggestions from the reviewers from reviewers commons.

There is still and open unanswered question about whether autophagic flux is occurring during the repair process.

Figure S1B/Figure 1: I t would be nice to see the quantification of the gH2AX staining for completeness in figure 1

Figure 2A: there is very little cytoplasmic co-localisation of LC3 and Lysotracker, so it is difficult to assess differences in autophagy during the drug treatment and recovery regime. I still think that either a western blot for LC3I and II accompanying figure 2A, or using the tandem LC3 construct, will provide stronger evidence that there is active autophagic flux removing the micronuclei. Failing that maybe inhibiting autophagy and then looking at the metrics of DNA damage in figure 1 would be an alternative approach.

Figure 3B, the arrowhead are in different locations in the figure in the manuscript and the figure in the response to reviewer. and it needs to described in the figure legend.

Check spelling throughout, there are a lot or lamina rather than lamin.

Reviewer 2

Advance summary and potential significance to field

Muciño-Hernandez and colleagues use of biochemical methodologies with confocal and super resolution microscopy to study the contribution of nucleophagy to genome stability. The authors suggest that nucleophagy contributes to the elimination of chromosomal fragments or nucleolar bodies exiting the nucleus upon DNA damage-inducing conditions. They propose a key role for nucleophagy in the maintenance of genome stability. The revised manuscript has been improved significantly. The authors performed additional experiments to address the comments of the Reviewers. However, several critical rigor issues remain. Some important questions are still left unaddressed. In addition, some new issues have emerged. Specifically:

© 2022. Published by The Company of Biologists under the terms of the Creative Commons Attribution License (https://creativecommons.org/licenses/by/4.0/).

Comments for the author

The key claim that autophagy has a causal role in formation and degradation of nuclear alterations need to be more robustly supported by data in the manuscript. In fact, basal and induced nuclear bond and micronuclei formation and degradation show different trends in control and autophagy deficient cells following etoposide treatment (revised Fig. 2). In addition, this part of the study is rather descriptive in nature and requires a better mechanistic explanation.

There are several issues with the new figure 2, particularly parts 2F and 2G:

The authors should also show the statistical comparisons between different timepoints, rather than just comparing control and siRNA / KO in the same timepoint. Why do the control cells in figures 2F and 2G exhibit so different behavior? Does the siRNA control have an effect? Why do the nuclear buds of the WT animals (2F) and the micronuclei of the siControl animals (2G) increase post recovery? Where is the evidence indicating that autophagy is necessary for the removal of micronuclei (as the subtitle of this section 210-211 suggests)? If anything, these graphs show enhanced removal of micronuclei post recovery when autophagy is inhibited.

The authors have not explained (and do not attempt to) how BECN1 initiates the formation of nuclear buds/micronuclei. They have only shown that ATG4 and ATG7 may have a role in the formation of these aberrations and that autophagy is most likely responsible for their clearance. Along similar lines, the varying degree of nuclear alterations observed in Atg4b-/- and Atg7-/- cells when compared with wild-type cells under various experimental conditions and the fact that the ATG4 family consists of four members (ATG4A, ATG4B, ATG4C and ATG4D), which can function redundantly in autophagosome formation, point to the need for elaborating on the role of autophagy in the nucleus.

Moreover, autophagic degradation of TOP2B should also be supported by additional experiments because the difference in TOP2B levels between control and Atg4 deficient cells, 5h after etoposide treatment is only marginal (Fig. 3H). In this panel, I suppose that TOP2B levels were quantified as a ratio to tubulin levels as shown in the Western blot and not to actin as written in the Y-axis of the graph on the right.

Neither the abundance of fibrillarin show any significant difference between wild-type and Atg4-deficient cells, as shown in the corresponding figure of rebuttal letter. Overall, the idea that fibrillarin and TO2B are autophagy substrates needs to be further investigated.

In Fig. 4B, there is no statistically significant difference in the percentage of control and etoposide-treated cells, with nuclear lesions containing fibrillarin and GFP-LC-3. In this case, the interpretation that “nucleolar DDR is a particular response independent of DDR elsewhere in the genome” needs to be explored.

Fig. 4D: In super resolution microscopy “not every nuclear alteration was found having GFP-LC3”: The above argument does not oppose the lack of GFP-LC3 enrichment at those structures. The authors have to explicitly show not only example(s) of what is considered as a GFP-LC3-positive structure but also what is considered as a GFP-LC3-negative structure. In any case, GFP-LC3 detection in “nuclear alterations containing Fibrillarin” is misleading when the non-“nuclear alterations containing Fibrillarin” parts of the cells have equal GFP-LC3 signal.

Figure 4E: In the presented image, 4 out of 7 cells and respective nuclei are devoid of FBL. Is this normal/anticipated? Could this be a technical problem with immunostaining?

Figure 4F and S3C: FBL band in IP:BECN1 lane seems somehow higher comparing to Input lane. Importantly, BECN1 immunoprecipitation is achieved (IP vs Input lane). However, FBL does not seem enriched in IP lane comparing to Input lane.

Although the authors discuss the evidence suggesting contribution of BECN1 to TOP2 nuclear extrusion, they should provide direct experimental support for this statement. Also, the fact that the authors do not detect a physical interaction between LC3 with either fibrillarin or TOP2A, nor

© 2022. Published by The Company of Biologists under the terms of the Creative Commons Attribution License (https://creativecommons.org/licenses/by/4.0/).

of TOP2B with BECN1 by immunoprecipitation needs to be further investigated. These are key points of the study.

As mentioned above, it appears that there is no direct interaction between LC3 and fibrillarin. If this is the case, it is plausible to assume that degradation of nuclear components under the experimental conditions tested is mediated by a putative autophagy receptor.

To support the notion that basal nucleophagy contributes to nuclear stability the authors should assess the survival of wild-type and autophagy deficient cells under their experimental conditions.

Unexpectedly, the authors removed Fig. S3 instead of performing statistical analysis to strengthen their hypothesis that nuclear alterations contain autophagic markers and TOP2B. I believe that the complete removal of Fig. S3 does not benefit the study. Graphs G and H in particular (TOP2B and BECN1) demonstrated a significant difference in behavior between nuclear buds and micronuclei, which supports the authors new claim about the two types of aberrations having potentially distinct mechanisms of formation and clearance.

Overall, the role of autophagy in extrusion and degradation of nuclear components under both physiological and DNA damage-induced conditions requires more in-depth investigation.

Minor points Figure 2F and S3A: Is ATG7 blot for siATG7 5h R available?

As requested, include protein marker to all blots, not only to the whole field blots in Figure S3.

Arrows are not well aligned in Figure 2A, D.

Zoomed areas are not accurate in Figure 2D and 3C.

<u>Reviewer 3</u>

Advance summary and potential significance to field

Peer review of the revised manuscript with the number RC-2021-01181 by Muciño-Hernandez G et. al. at Journal of Cell Science and with the tittle "Nucleophagy contributes to genome stability though TOP2cc and nucleolar components degradation"

After reviewing the files of the revised version of the manuscript by Muciño-Hernandez G et. al. I confirm my positive opinion about the work. The authors show in their revised manuscript that mouse embryonic fibroblasts (MEFs) have basal levels of nuclear buds and micronuclei, which are indicators of genomic DNA damage. These basal levels of nuclear buds and micronuclei in MEFs increased after Etoposide treatment, which is known to induce DNA Double stranded Breaks (DSD). Interestingly, the nuclear buds and micronuclei co-localize with makers for nucleophagy (BECN1 and LC3) and acidic vesicles, suggesting that they are cleared by nucleophagy. The authors propose that basal levels of nucleophagy clear basal levels of genomic DNA damage that occurs as result from DNA-dependent biological processes in the cell nucleus, thereby contributing to nuclear stability of MEFs under physiological conditions. These basal levels of nucleophagy increase after the action of factors that induce DNA damage and nuclear stress. The concepts proposed by Muciño-Hernandez G et. al. are novel since most of the current published data on nucleophagy related to DNA damage have been obtained under pathological conditions.

The authors have answered to the concerns raised by the reviewers in scientifically sound manner. The authors have included into the revised manuscript new data from experiments that were performed following the suggestions of the reviewers. In addition, the authors improved the Figures, the description of the Figures in the Results section, as well as the availability of the results by providing the Table S1. In general, the data support the claims of the authors. The quality of the data is sound. The structure of the manuscript supports the understanding of the reader. Summarizing, the revised version of the manuscript by Muciño-Hernandez G et. al. fits to the standards of articles recently published at the Journal of Cell Science. I hope that the other reviewers and the editorial staff from the Journal of Cell Science share my positive view about the

© 2022. Published by The Company of Biologists under the terms of the Creative Commons Attribution License (https://creativecommons.org/licenses/by/4.0/).

novelty and quality of the manuscript by Muciño-Hernandez G et. al. and recommend it for publication as Article at the Journal of Cell Science.

Comments for the author

After reviewing the files of the revised version of the manuscript by Muciño-Hernandez G *et. al.* I confirm my positive opinion about the work. The authors show in their revised manuscript that mouse embryonic fibroblasts (MEFs) have basal levels of nuclear buds and micronuclei, which are indicators of genomic DNA damage. These basal levels of nuclear buds and micronuclei in MEFs increased after Etoposide treatment, which is known to induce DNA Double stranded Breaks (DSD). Interestingly, the nuclear buds and micronuclei co-localize with makers for nucleophagy (BECN1 and LC3) and acidic vesicles, suggesting that they are cleared by nucleophagy. The authors propose that basal levels of nucleophagy clear basal levels of genomic DNA damage that occurs as result from DNA-dependent biological processes in the cell nucleus, thereby contributing to nuclear stability of MEFs under physiological conditions. These basal levels of nucleophagy increase after the action of factors that induce DNA damage and nuclear stress. The concepts proposed by Muciño-Hernandez G *et. al.* are novel, since most of the current published data on nucleophagy related to DNA damage have been obtained under pathological conditions.

The authors have answered to the concerns raised by the reviewers in scientifically sound manner. The authors have included into the revised manuscript new data from experiments that were performed following the suggestions of the reviewers. In addition, the authors improved the Figures, the description of the Figures in the Results section, as well as the availability of the results by providing the Table S1. In general, the data support the claims of the authors. The quality of the data is sound. The structure of the manuscript supports the understanding of the reader. Summarizing, the revised version of the manuscript by Muciño-Hernandez G *et. al.* fits to the standards of articles recently published at the Journal of Cell Science. I hope that the other reviewers and the editorial staff from the Journal of Cell Science share my positive view about the novelty and quality of the manuscript by Muciño-Hernandez G *et. al.* and recommend it for publication as Article at the Journal of Cell Science.

First revision

Author response to reviewers' comments

Point by point response.

Reviewer 1

The manuscript is signficantly improved with the suggestions from the reviewers from reviewers commons. There is still and open unanswered question about whether autophagic flux is occurring during the repair process.

Figure S1B/Figure 1: I t would be nice to see the quantification of the gH2AX staining for completeness in figure 1
Thank you for the suggestion. We quantified 48 cells per experiment, in three independent experiments. The results are now included in the revised Figure 1D.

Figure 2A: there is very little cytoplasmic co-localisation of LC3 and Lysotracker, so it is difficult to assess differences in autophagy during the drug treatment and recovery regime.
In the original figure we pointed out only micronuclei containing DNA and labeled by Lysotracker and having LC3 or BECN1. In the revised version we now point out also vesicles with Lysotracker signal next to LC3 labeled vesicles, suggesting their are about to fuse. We also indicate some LC3 labeled vesicles with Lysotracker inside, indicating autophagosome-lysosomes fusion. The abundance of such vesicles is higher after 2h of DNA damage, and are reduced after 5 hr of recovery, which indicates a proper autophagic flux. If it were stalked, we would observe a constant or even higher amount of independent Lysotracker staining and LC3 labeled vesicles after 5 hr of recovery.

© 2022. Published by The Company of Biologists under the terms of the Creative Commons Attribution License (https://creativecommons.org/licenses/by/4.0/).

I still think that either a western blot for LC3I and II accompanying figure 2A, or using the tandem LC3 construct, will provide stronger evidence that there is active autophagic flux removing the micronuclei. Failing that maybe inhibiting autophagy and then looking at the metrics of DNA damage in figure 1 would be an alternative approach.
Thank you for your suggestions. Following your advice, we compared the level of DNA damage and repair in cells treated or not with Spautin1, an inhibitor of autophagy (Cell (2011) **147**(1): 223-234). We observed that when autophagy is inhibited the level of DNA damage is statistically significant higher after 2h of Etoposide treatment, and it is not completely repaired after 5 hr of Etoposide removal. These results suggest that autophagy contributes to maintain DNA stability in our model. We included these results in the new Figure 1C.

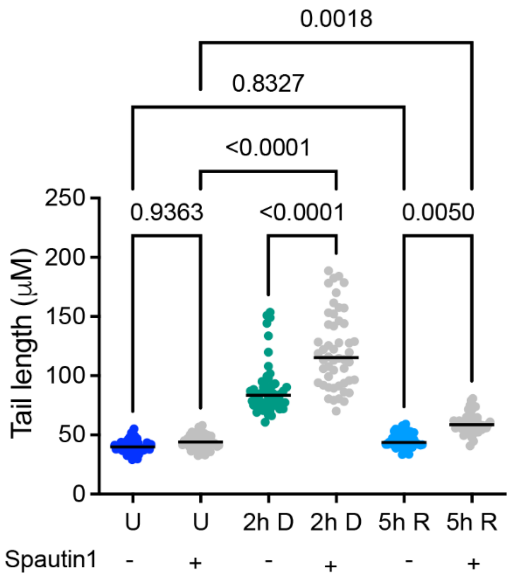

Figure 1. Comet assay to quantify the level of DNA damage in untreated cells (U), after 2 h of Etoposide exposure (2h D), and after 5h of Etoposide removal (5h R), with DMSO as vehicle (-) or 10 µM Spuatin1 12 hr before Etoposide treatment (+). Bars represent median at each time point, statistical significant differences were determined by Two-way ANOVA followed by Tukey´s multiple comparison test; adjusted P value is indicated for each comparison. 50 comets were measured in each of three independent experiments.

Figure 3B, the arrowhead are in different locations in the figure in the manuscript and the figure in the response to reviewer. and it needs to described in the figure legend.
Thank you, we corrected the position of the arrowheads and the text.

Check spelling throughout, there are a lot or lamina rather than lamin.
We apologize for those errors. We checked the document and corrected the mistakes found.

Reviewer 2

The key claim that autophagy has a causal role in formation and degradation of nuclear alterations need to be more robustly supported by data in the manuscript. In fact, basal and induced nuclear bond and micronuclei formation and degradation show different trends in control and autophagy deficient cells following etoposide treatment (revised Fig. 2). In addition, this part of the study is rather descriptive in nature and requires a better mechanistic explanation.

There are several issues with the new figure 2, particularly parts 2F and 2G:
The authors should also show the statistical comparisons between different timepoints, rather than just comparing control and siRNA / KO in the same timepoint.
Following the reviewer´s advice, in the revised manuscript we now include a multiple comparison analysis among treatments and time points, shown in the revised Figures S3B and S3C (for space limitation in the main figure 2), displaying adjusted P values calculated by 2-Way Anova followed by Tukey´s multiple comparison test.

© 2022. Published by The Company of Biologists under the terms of the Creative Commons Attribution License (https://creativecommons.org/licenses/by/4.0/).

Why do the control cells in figures 2F and 2G exhibit so different behavior? Does the siRNA control have an effect?

Control siRNA does not seem to have an effect. The behavior of control cells across experiments is rather similar. If we compare the percentage of cells with nuclear buds in untreated cells transfected with control siRNA (blue triangles in (U) in figure 2F) and the percentage of cells with nuclear buds of untreated *Atg4*$^{+/+}$ cells (blue triangles in control (U) in figure 2G) there is little difference. While on average 19.8% of the control cells transfected with siRNA control had nuclear buds (Fig2F), 17.98% of wild type untreated cells had buds (fig 2F). If we compare the percentage of cells with micronuclei, we observe they are also similar: 3.8% of untreated cells transfected with siRNA control (Fig 2F) had micronuclei, while 4.63% of untreated WT cells had them (fig 2G).

Why do the nuclear buds of the WT animals (2F) and the micronuclei of the siControl animals (2G) increase post recovery?

The percentage of WT cells with nuclear buds does not increase post recovery (Fig 2G), but it is true that we observed a contrasting behavior in untreated cells transfected with control siRNA, where the percentage of cells having buds increased with statistically significance when compared to untreated cells and after 2 hr of DNA damage (Fig 2F and new Fig S2B). It is not an effect of the control siRNA transfected, because we observed the same response in untransfected cells (Fig 1F).

Where is the evidence indicating that autophagy is necessary for the removal of micronuclei (as the subtitle of this section 210-211 suggests)? If anything, these graphs show enhanced removal of micronuclei post recovery when autophagy is inhibited.

Thank you for this observation. We meant that autophagy contributes to micronuclei removal during DNA damage, not after recovery.

When we compared the percentage of cells with micronuclei in WT *vs.* *Atg4*$^{-/-}$ cells, we observed that even in untreated cells when autophagy is compromised the number of cells with micronuclei shows a trend to double (Fig 2G, 4.63% of WT cells vs 8.11% of *Atg4*$^{-/-}$ cells; p=0.55). In response to DNA damage while 5.1% of the WT cells have micronuclei, 13.9% of *Atg4*$^{-/-}$ cells have micronuclei. Therefore, a) the biogenesis of micronuclei occurs in the absence of functional autophagy, and b) the fact that there are more cells with micronuclei in *Atg4*$^{-/-}$ than in WT upon DNA damage, indicates that autophagy contributes to their removal. It is true that after 5 hr of recovery there is no difference in the percentage of cells with micronuclei between WT and *Atg4*$^{-/-}$ cells. We failed to expressed it in the manuscript. In the current version we express our results with more caution.

The authors have not explained (and do not attempt to) how BECN1 initiates the formation of nuclear buds/micronuclei. They have only shown that ATG4 and ATG7 may have a role in the formation of these aberrations and that autophagy is most likely responsible for their clearance. Along similar lines, the varying degree of nuclear alterations observed in Atg4b-/- and Atg7-/- cells when compared with wild-type cells under various experimental conditions and the fact that the ATG4 family consists of four members (ATG4A, ATG4B, ATG4C and ATG4D), which can function redundantly in autophagosome formation, point to the need for elaborating on the role of autophagy in the nucleus.

Based on our observations and published data, we imagine an integrative role of BECN1 in the simultaneous regulation of DNA damage repair and autophagosome formation as part of the DNA damage response. A nuclear localization of BECN1 has been also documented by others, it shuttles between nucleus and cytoplasm to participate in different functions, such as DNA damage repair through an interaction with TOP2B, and autophagosome formation (Xu F. et al. (2017) Sci Rep 7:45385; Liang XH, et al. (2001) Cancer Res 61(8): 3443-9). As it is shown in figures 2A and S2, previous to DNA damage, BECN1 is located mainly in the nucleus, interestingly in specific nuclear bodies. Upon DNA damage it exits the nucleus and becomes distributed throughout the cell, and when DNA is repaired BECN1 localizes again at specific nuclear bodies. As shown in figure 2C, an important proportion of nuclear alterations contain BECN1. A tempting speculation is that BECN1 could drive the expulsion of nuclear damaged material into the cytoplasm. Since we observed that BECN1 is exported to the cytoplasm during DNA damage, and that BECN1 exits the nucleus interacting with TOP2B, perhaps BECN1 helps nuclear damaged material to be expelled from the nucleus and recognized by the autophagy machinery. Whether this is true and the exact mechanism explaining how, requires further investigations beyond the scope of the current manuscript.

© 2022. Published by The Company of Biologists under the terms of the Creative Commons Attribution License (https://creativecommons.org/licenses/by/4.0/).

Moreover, autophagic degradation of TOP2B should also be supported by additional experiments because the difference in TOP2B levels between control and Atg4 deficient cells, 5h after etoposide treatment is only marginal (Fig. 3H).
Since we observed by immunofluorescence that only 5-10% of the cells develop nuclear alterations containing TOP2B at the time points studied, it is not surprising that pooling together all the cells in a Western blot dilutes the potential autophagic degradation of TOPB. Also, the amount of TOP2B located inside micronuclei is rather small compared to the total amount of TOP2B in the cell. Taking into account these two observations, the marginal reduction observed by Western blot is rather supportive.

In this panel, I suppose that TOP2B levels were quantified as a ratio to tubulin levels as shown in the Western blot and not to actin as written in the Y-axis of the graph on the right.
This is correct. We apologize for the mistake. We corrected it in the current version of the manuscript.

Neither the abundance of fibrillarin show any significant difference between wild-type and Atg4-deficient cells, as shown in the corresponding figure of rebuttal letter. Overall, the idea that fibrillarin and TO2B are autophagy substrates needs to be further investigated.
The percentage of cells having nuclear alterations containing FBL, and the small proportion of FBL in such nuclear alterations, make it difficult to demonstrate by Western blot the autophagic degradation of such a small proportion of FBL with respect to total FBL in the cells. Single cell analysis are required. We agree with the reviewer. Further experiments are needed, beyond the scope of the current manuscript.

In Fig. 4B, there is no statistically significant difference in the percentage of control and etoposide-treated cells, with nuclear lesions containing fibrillarin and GFP-LC-3. In this case, the interpretation that "nucleolar DDR is a particular response independent of DDR elsewhere in the genome" needs to be explored.
We agree with the reviewer, but exploring nucleolar DDR is beyond the scope of the current work.

Fig. 4D: In super resolution microscopy "not every nuclear alteration was found having GFP-LC3": The above argument does not oppose the lack of GFP-LC3 enrichment at those structures. The authors have to explicitly show not only example(s) of what is considered as a GFP- LC3-positive structure but also what is considered as a GFP-LC3- negative structure. In any case, GFP-LC3 detection in "nuclear alterations containing Fibrillarin" is misleading when the non-"nuclear alterations containing Fibrillarin" parts of the cells have equal GFP-LC3 signal.
We used super resolution microscopy to evaluate the localization of both GFP-LC3 and FBL en the same location in the cells. An example of nuclear alterations with FBL lacking GFP-LC3 quantified in Fig 4B is shown in the next figure:

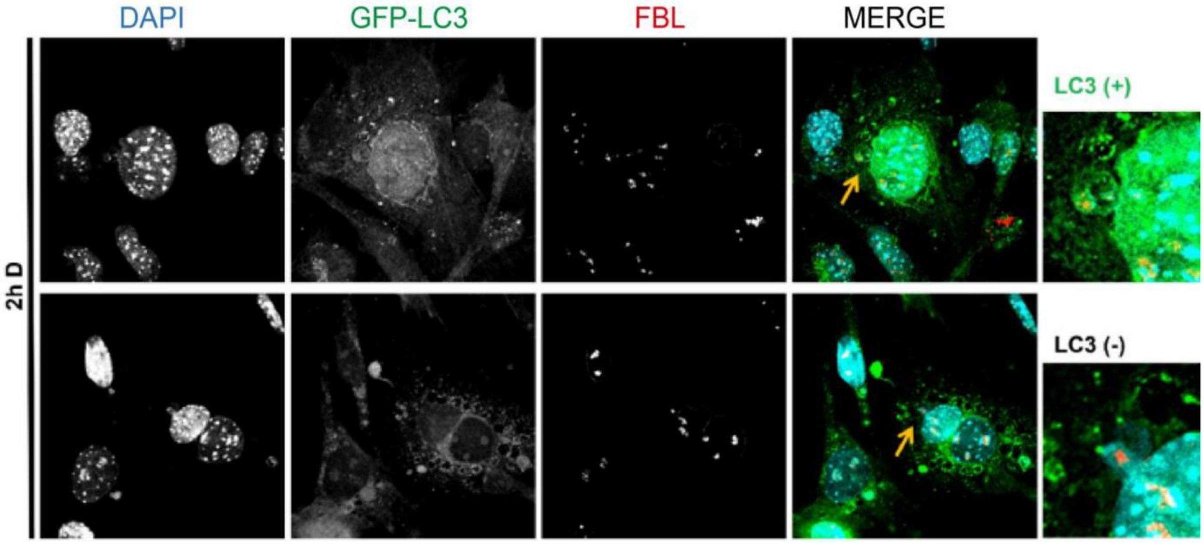

© 2022. Published by The Company of Biologists under the terms of the Creative Commons Attribution License (https://creativecommons.org/licenses/by/4.0/).

Figure 1. Arrows indicate nuclear buds positive for DAPI and FBL. In the upper row we show an example of of a micronuclei having also GFP-LC3, labeled LC3(+). In the bottom row we show an example of a micronuclei having FBL but no GFP-LC3, labeled LC3(-).

Figure 4E: In the presented image, 4 out of 7 cells and respective nuclei are devoid of FBL. Is this normal/anticipated? Could this be a technical problem with immunostaining?
Yes, in order to detect simultaneously BECN1 and FBL, we had to used an antibody to detect FBL that gave faint signals in several cells. We increased the exposure in the revised figure 4E.

Figure 4F and S3C: FBL band in IP:BECN1 lane seems somehow higher comparing to Input lane. Importantly, BECN1 immunoprecipitation is achieved (IP vs Input lane). However, FBL does not seem enriched in IP lane comparing to Input lane.
If only a fraction of the FBL molecules inside the cells are interacting with BECN1, as expected because only a fraction of nucleolar components would be targeted to nuclear alterations, the amount of FBL co-immunoprecipitated with BECN1 should be smaller than the amount of FBL observed in a total extract.

Although the authors discuss the evidence suggesting contribution of BECN1 to TOP2 nuclear extrusion, they should provide direct experimental support for this statement.
We demonstrated that TOP2B in found in nuclear alterations containing also BECN1, and found some cells with a bridge joining the main nucleus with a micronuclei, containing both BECN1 and TOP2B. This qualitative observation suggests that BECN1 is involved in the exclusion of nuclear material. Further experiments to evaluate the formation of nuclear alterations in the absence of BECN1 would be a direct proof of the role of BECN1. Such experiments are beyond de scope of the present manuscript.

Also, the fact that the authors do not detect a physical interaction between LC3 with either fibrillarin or TOP2A, nor of TOP2B with BECN1 by immunoprecipitation needs to be further investigated. These are key points of the study.
We found that TOP2B is surrounded by LC3 by co-immunogold localization, detected by transmission electron microscopy, and we actually catch them in some cells exiting the nucleus together. The fact that we did not confirm an interaction by co-IP suggest that those complexes are not abundant. Some biological process are lost when using techniques where all the cells are pulled together and all the intracellular structures eliminates, such as in the lysis needed to do the IP. Single cell analysis are needed to further investigate the molecular mechanism of nucleopahgy, which are beyond the scope of the current manuscript.

As mentioned above, it appears that there is no direct interaction between LC3 and fibrillarin. If this is the case, it is plausible to assume that degradation of nuclear components under the experimental conditions tested is mediated by a putative autophagy receptor.
Yes, we think there should be an autophagic receptor that recognizes nuclear damaged components. While cGAS has been proposed as a nucleophagy receptor, it does so for nacked cytoplasmic DNA. Since we observed micronuclei (*i.e.* DNA protected by micronuclear envelop), a different molecule should be detecting it. The identification of such receptor is extremely interesting, but beyond the scope of this manuscript.

To support the notion that basal nucleophagy contributes to nuclear stability, the authors should assess the survival of wild-type and autophagy deficient cells under their experimental conditions.
We assessed the survival and it was above 90% in every condition.

Unexpectedly, the authors removed Fig. S3 instead of performing statistical analysis to strengthen their hypothesis that nuclear alterations contain autophagic markers and TOP2B. I believe that the complete removal of Fig. S3 does not benefit the study. Graphs G and H in particular (TOP2B and BECN1) demonstrated a significant difference in behavior between nuclear buds and micronuclei, which supports the authors new claim about the two types of aberrations having potentially distinct mechanisms of formation and clearance.
Thank you for this observation. We now include again such analysis.

Overall, the role of autophagy in extrusion and degradation of nuclear components under both physiological and DNA damage-induced conditions requires more in-depth investigation.

© 2022. Published by The Company of Biologists under the terms of the Creative Commons Attribution License (https://creativecommons.org/licenses/by/4.0/).

We agree with the reviewer. Future work is needed, beyond the scope of the current manuscript.

Minor points

Figure 2F and S3A: Is ATG7 blot for siATG7 5h R available?
Unfortunately not, bu we have observed that the silencing effect of siRNAs normally last 48 hr or more.

As requested, include protein marker to all blots, not only to the whole field blots in Figure S3.
Thank you for your recommendation. All molecular markers are indicated in the current version.

Arrows are not well aligned in Figure 2A, D.
We corrected the position of the arrows.

Zoomed areas are not accurate in Figure 2D and 3C.
Thank you for this observations. We corrected them.

Reviewer 3

The authors have answered to the concerns raised by the reviewers in scientifically sound manner. The authors have included into the revised manuscript new data from experiments that were performed following the suggestions of the reviewers. In addition, the authors improved the Figures, the description of the Figures in the Results section, as well as the availability of the results by providing the Table S1. In general, the data support the claims of the authors. The quality of the data is sound. The structure of the manuscript supports the understanding of the reader. Summarizing, the revised version of the manuscript by Muciño-Hernandez G et. al. fits to the standards of articles recently published at the Journal of Cell Science. I hope that the other reviewers and the editorial staff from the Journal of Cell Science share my positive view about the novelty and quality of the manuscript by Muciño-Hernandez G et. al. and recommend it for publication as Article at the Journal of Cell Science.

We are very thankful to reviewer 3.

Second decision letter

MS ID#: JOCES/2022/260563

MS TITLE: Nucleophagy contributes to genome stability though TOP2cc and nucleolar components degradation

AUTHORS: Gabriel Mucino-Hernandez, Pilar S Acevo-Rodriguez, Sandra Cabrera, Adan Oswaldo Guerrero, Horacio Merchant-Larios, and Susana Castro-Obregon
ARTICLE TYPE: Research Article

Thank you for sending your manuscript to Journal of Cell Science through Review Commons.

I am happy to tell you that your manuscript has been accepted for publication in Journal of Cell Science, pending standard ethics checks.

© 2022. Published by The Company of Biologists under the terms of the Creative Commons Attribution License (https://creativecommons.org/licenses/by/4.0/).
